# A Sulfonic Acid Polyvinyl Pyridinium Ionic Liquid Catalyzes the Multi-Component Synthesis of Spiro-indoline-3,5′-pyrano[2,3-*d*]-pyrimidines and -Pyrazines

**DOI:** 10.3390/molecules28093663

**Published:** 2023-04-23

**Authors:** Mehdi Khalaj, Mahboubeh Taherkhani, Leo Payen, Axel Klein

**Affiliations:** 1 Department of Chemistry, Buinzahra Branch, Islamic Azad University, Buinzahra 1477893855, Iran; 2 Department of Chemistry, Takestan Branch, Islamic Azad University, Takestan 3481949479, Iran; 3 Institute for Inorganic Chemistry, Department of Chemistry, Faculty of Mathematics and Natural Sciences, University of Cologne, Greinstrasse 6, 50939 Köln, Germany

**Keywords:** polymeric ionic liquid, polyvinyl pyridine, spiro-indoline-3,5′-pyrano[2,3-*d*]pyrimidine, spiro-indoline-3,4′-pyrano[2,3-*c*]pyrazole, ultrasonic

## Abstract

A sulfonated poly-4-vinyl pyridinium (PVPy-IL-B-SO_3_H) containing an acidic pyridinium/HSO_3_^−^ ionic liquid moiety was prepared and used as a catalyst for the three-component reaction of malononitrile with 1-alkylindoline-2,3-diones and 1,3-dimethylpyrimidine-2,4,6(1*H*,3*H*,5*H*)-trione or methyl 5-hydroxy-1*H*-pyrazole-3-carboxylate, leading to methyl 6′-amino-5′-cyano-2-oxo-2′*H*-spiro[indoline-3,4′-pyrano[2,3-*c*]pyrazole]-3′-carboxylates or -3,4′-pyrano[2,3-*d*]pyrimidine]-6′-carbonitrile derivatives under ultrasonic irradiation conditions. The solid catalyst allows easy separation, is cheap, produces high yields under mild conditions, and does not require column chromatography for product isolation and purification.

## 1. Introduction

**Scheme 1:**
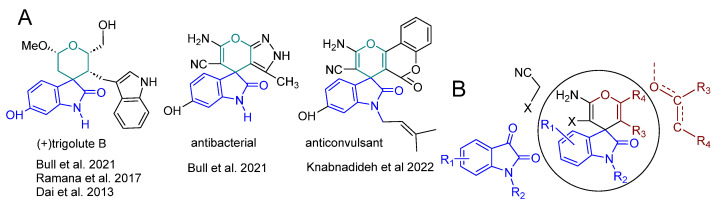
(**A**) Selected spirooxindole-pyranes and (**B**) three-component reaction to form them. Adopted from [3,9,10,11].

Recent advances in the synthetic chemistry of spirooxindoles in recent years have made this interesting substance class available for drug development in various medical fields such as antibiotics and cancer [1,2,3,4,5,6,7,8]. Among them, spirooxindole-pyranes are not part of established drugs. However, the tetrahydropyrane (*+*)trigolute B (Scheme 1A) was found as a part of six alkaloids in extracts from the plant *Trigonostemon lutescens*. It showed promising activity against hemorrhagic fever and weak acetylcholinesterase inhibition [9,10]. This has triggered enormous synthesis efforts to make the general class of spirooxindole pyranes available for drug development [1,2,3,4,5,6,7,8,11,12,13]. Recently, spiro-indoline-3,4′-pyrano[2,3-*c*]pyrazoles were also tested as corrosion inhibitors [14]. One option for the synthesis of spiroindoline-3,4′-pyranes is the three-component reaction of 1-alkylindoline-2,3-diones with malonitrile derivatives and an activated enolate (Scheme 1B) [2,4,5,6,8,11,12,13,15,16,17,18,19,20,21,22]. Using such procedures, reactions of *N*-alkyl-1-(methylthio)-2-nitroethenamines, barbituric acid, and isatin (1*H*-indole-2,3-dione) in water produced spiroindoline-3,4′-pyrano[2,3-*c*]pyrimidines [15], the three-component reaction of barbituric acids, isatin, and cyclohexane-1,3-diones catalyzed by *para*-toluene sulfonic-acid-produced spiro[chromeno[2,3-*d*]pyrimidine-5,3-indoline]-tetraones [16], and spiroindoline-3,4′-pyrano[2,3-*c*]naphthalenes were obtained from isatin or its *N*-methyl derivative and 1- or 2-naphthol in reactions catalyzed by InCl_3_ under microwave conditions [21].

Further strategies include 1,3-dipolar cycloadditions, multi-component cyclizations, and Michael additions followed by cyclizations [1,3,4,5,6,7,13,14,23,24]. The four-component reaction of phenylhydrazines, dialkyl acetylenedicarboxylate, substituted isatin (2,3-indolindion) derivatives, and malononitrile catalyzed by *L*-proline under ultrasonic irradiation condition produced spiro-indoline-3,4′-pyrano[2,3-*c*]pyrazole derivatives [23]. Furthermore, four-membered [4 + 1] and formal [4 + 2] cycloaddition reactions were reported to produce a wide range of spirooxindole derivatives, including pyranes [5,6,13,24].

**Scheme 2:**
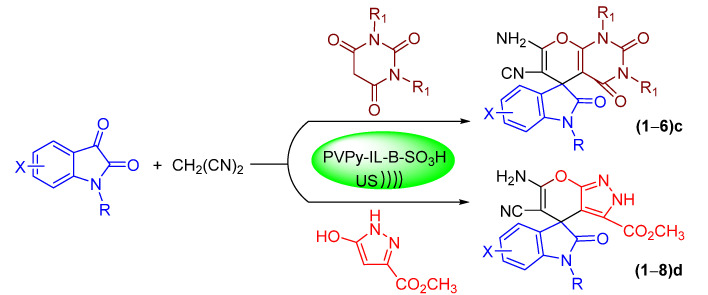
Synthesis pathways for the products (**1**–**6**)**c** and (**1**–**8**)**d**. R = Et, *n*-Pr, Bn, EtPh; R_1_ = Me, Et, Bn; X = H, 5-Me, 5-OMe, 5-Cl, 5-Br, 5,7-Me_2_, 5,7-Cl_2_, 5,7-Br_2_.

In continuation of our ongoing research [25,26,27,28,29], we herein report on the synthesis of a poly(4-vinyl-pyridinium-*N*-butyl-sulfonate) material (PVPy-IL-B-SO_3_H) containing an acidic pyridinium/HSO_3_^−^ ionic liquid moiety and its use as an acid catalyst for the preparation of the spirooxoindoles **1c**–**6c** and **1d**–**8d** (Scheme 2) through the three-component reaction of malononitrile with 1-alkylindoline-2,3-diones and 1,3-dialkylpyrimidine-2,4,6(1*H*,3*H*,5*H*)-triones or methyl 5-hydroxy-1*H*-pyrazole-3-carboxylate under ultrasonic irradiation (US) conditions.

Polymers with acidic protons in their structures and ionic liquid-polymer composites have found applications in fuel cells, in biomedical applications, and as catalysts [30,31,32,33,34] and different methods such as grafting, crosslinking, and functionalization procedures have been developed to produce such materials. Our idea of using an acidic polymer catalyst based on poly(4-vinyl pyridine) was motivated by the wish to find a heterogenized (supported) and thus easily removable acidic catalyst for the versatile reactions shown in Scheme 2. Similar approaches using “Carbon-SO_3_H” [20], “silica-sulfuric acid nanoparticles” [19], magnetically removable Fe_3_O_4_@SiO_2_-SO_3_H and Fe_3_O_4_@SiO_2_[(CH_2_)_3_-DABCO-SO_3_H]Cl_2_ nanocatalysts [7], H_2_SO_4_-functionalized graphitic carbon nitride [35], and sulfonated nanoporous silica (SBA-15-Pr-SO_3_H) [36] for the synthesis of spirooxindole pyranes were previously reported. We have recently used a similar solid acid catalyst, di(3-propylsulfonic acid grafted on NiFe_2_O_4_@SiO_2_ for the synthesis of chromeno[4,3-*d*]pyrido[1,2-*a*]pyrimidine derivatives [26].

## 2. Results and Discussion

### 2.1. Catalyst Preparation and Characterization

**Scheme 3:**
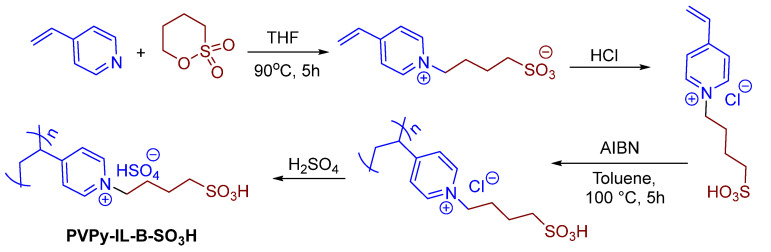
Preparation of PVPy-IL-B-SO_3_H.

The catalyst PVPy-IL-B-SO_3_H was prepared as shown in Scheme 3. First, 4-vinylpyridine was reacted with 1,2-oxathiane-2,2-dioxide leading to 4-(4-vinylpyridinium-1-yl)butane-1-sulfonate which represents a type of ionic liquid. Acidification with hydrochloric acid caused the formation of 1-(4-sulfobutyl)-4-vinylpyridinium chloride. This compound was polymerized using azobisisobutyronitrile (AIBN) and the final product was obtained by reaction of the polymer with sulfuric acid (for details, see Materials and Methods). Elemental analysis produced 39.25% C, 5.49% H, 4.04% N, and 17.98% S. Calculated values for complete pyridine–*n*-Bu-SO_3_H alkylation and Cl^−^ to ^−^SO_3_H replacement (C_11_H_17_NO_7_S_2_, M_W_ = 339.38 g/mol, see Scheme 3) are C, 38.93; H, 5.05; N, 4.13; and S, 18.89%. From the N values, we calculated a 97% match with the ideal catalyst formula shown in Scheme 3; from the S values it was 95%. The barium sulfate test showed that the sample contained a total of 5.06 mmol of SO_4_^2−^ ions per gram, which was equal to 5.06 mmol H^+^/g in the catalyst.

**Figure 1:**
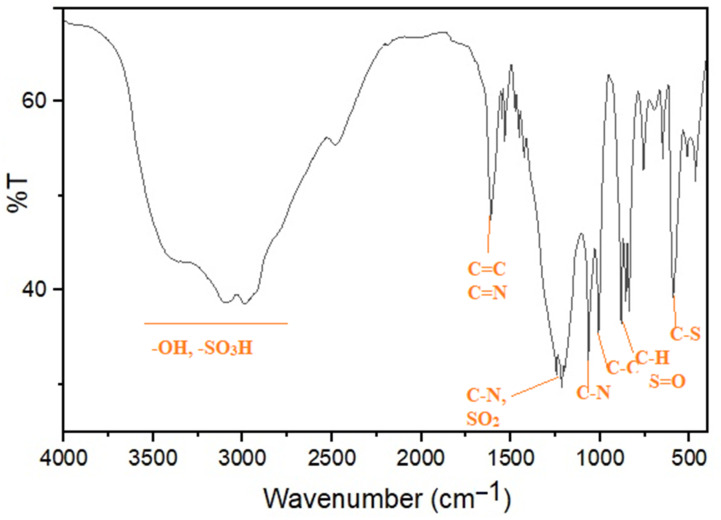
FT-IR spectrum of PVPy-IL-B-SO_3_H.

The Fourier Transform IR (FT-IR) spectrum (Figure 1) of the as-prepared PVPy-IL-B-SO_3_H showed a broad intense band at 2500–3500 cm^−1^, which was assigned to C–H stretches of the hydrocarbon groups and O–H stretches of the SO_3_H group and attached H_2_O molecules [26], a sharp resonance at 1613 cm^−1^ assigned to the C=C and C=N stretching vibrations of the pyridyl group, and bands at 1247 (C–N stretching), 1215 (SO_2_ asymmetric stretching), 1067 (C–N stretching), 1013 (C–C, and C–S stretching), 883 (C–H bending, S=O stretching), and 597 cm^−1^ (C–S bending).

**Figure 2:**
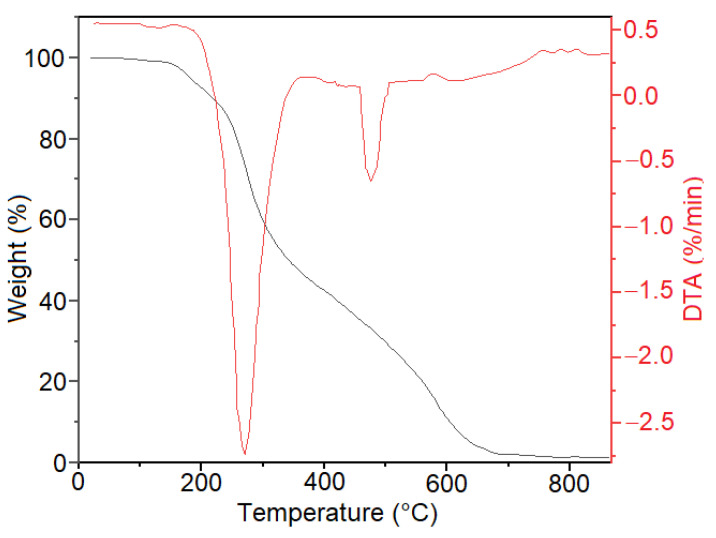
TGA-DTA of PVPy-IL-B-SO_3_H under an N_2_ atmosphere.

A thermogravimetric analysis (TGA) of PVPy-B-SO_3_H showed two main stages of weight loss (Figure 2). The first stage occurred from 200 to 400 °C with an approximate weight loss of about 50%, which might be related to the splitting of the sulfonic acid butyl groups. The second stage of weight loss of another 50% from 400 to 600 °C was probably related to the destruction and degradation of the polymer structure. The differential thermal analysis (DTA) showed two major endothermic peaks at 270 °C, probably corresponding to the cleavage of SO_2_ or SO_3_ and at 475 °C, indicating CO_2_ release during polymer degradation (Figure 2).

**Figure 3:**
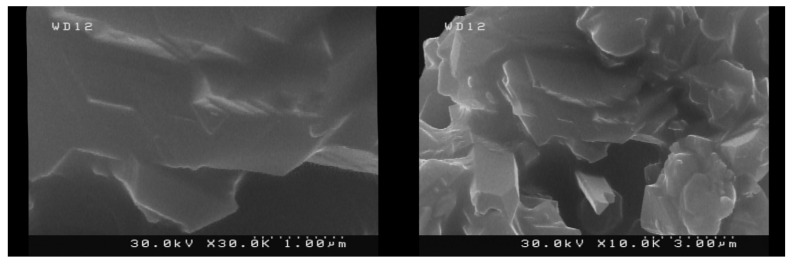
FE-SEM photographs of PVPy-IL-B-SO_3_H. Scale bars are 1 µm (**left**) and 3 µm (**right**).

Field-emission scanning electron microscopy (FE-SEM) showed agglomerated platelets with diameters in the lower µm range (Figure 3).

### 2.2. Synthesis of Spiro-Indoline-3,5′-pyrano[2,3-*d*]pyrimidines—Optimization

The catalytic potential of PVPy-IL-B-SO_3_H was studied under ultrasonic irradiation (US) conditions in the three-component model reaction of malononitrile (1 mmol), 1-ethylindoline-2,3-dione (1 mmol), and 1,3-dimethylpyrimidine-2,4,6(1*H*,3*H*,5*H*)-trione (1 mmol) leading to the pyrimidine **1c**. The reaction was optimized by varying catalyst amount, solvent, and temperature (Table 1). The progress of the reaction was monitored by thin-layer chromatography (TLC) and driven to full conversion in each case. After complete conversion, the catalyst was removed by filtration and the product was isolated from the filtrate. The ^1^H and ^13^C NMR are shown in Figures S1 and S2, Supplementary Material.

A yield of 95% was obtained using a 0.04 g catalyst in EtOH under reflux for 50 min (Table 1, entry 4). A higher amount of catalyst (0.06 g) and a slightly shorter reaction time (45 min) produced the same result (entry 6). Non-polar solvents such as diethyl ether (Et_2_O), *n*-hexane, toluene, ethyl acetate (EtOAc) and CH_2_Cl_2_ were unsuitable and produced no conversion, while the polar solvents DMF (23%), CH_3_CN (48%), and H_2_O (45%) produced low yields. Reactions without a catalyst produced no conversion (entry 1), and lower *T* than reflux (78 °C) produced lower yields (entries 2 and 3).

In a previous study, isatin was reacted with malonitrile and 1,3-dimethylpyrimidine-2,4,6(1*H*,3*H*,5*H*)-trione using oleic acid as a homogeneous acid catalyst, and the NH (instead of NEt) derivative of **1c** was obtained in an 89% yield in 30 min [22]. The best results for the four-component reaction of indoline-2,3-dione and pyrimidine-2,4,6(1*H*,3*H*,5*H*)-trione with methylamine and nitroketene dithioacetals to 7′-(methylamino)-6′-nitrospiro[indoline-3,5′-pyrano[2,3-*d*]pyrimidine]-2,2′,4′(1′*H*,3′*H*)-trione similar to **1c** (NO instead of CN, MeNH instead of NH_2_ on the pyrane, and NH instead of NMe on the pyrimidine) were obtained without any catalyst. The components were heated 7 h in water and a yield of 72% was obtained [4,15]. Mixing EtOH to the water produced essentially the same yield (70%). Shorter reaction times of 24 h were obtained in EtOH under reflux but produced only a 65% yield. Even lower times of 3 h required base catalysts as piperidine or Et_3_N and produced only a 59 and 54% yield, respectively [15]. Using no solvents (neat) and a sulfonated silica catalyst for the above-mentioned product (NH instead of NEt) produced an excellent yield of 91% and a reaction time of 40 min [36]. These results are in line with our finding that EtOH is an ideal solvent for this reaction. Further, this comparison shows that our catalyst shows high activity in conjunction with the ultrasonic conditions. Furthermore, a series of syntheses starting from isatin, malonitrile, and various 1,3-diketones clearly showed the necessity of a catalyst, here citric acid, to come to reasonable reaction times (less than 2 h) and good yields (higher than 90%) [12].

When making an overall comparison over the so-far reported methods for this type of reactions, it first becomes clear that acid catalysts are superior to bases in terms of reaction times [4,7,22], and both are superior to procedures without any added catalyst [4,7,12,15,21,22]. The use of light to initiate such reactions has turned out to be not as efficient as acid-catalyzed reactions. Syntheses comparable to ours required reaction times from 4 to 12 h at ambient temperature and 18 W while light LEDs [13]. Highly efficient recoverable catalysts have been developed [4,7,19,20,35,36,37,38], including SO_3_H functionalized nanomaterials and polymers [4,7,19,20,35,36], 1-(2-aminoethyl)piperazine modified graphene oxide material [11], and L-proline-melamine polymers [38]. They are generally easier to separate than homogeneous catalysts. Even the simple Lewis acid salts such as CaCl_2_ has been successfully used [39]. As in our case, some approaches have also used ultrasound to activate the solid catalysts and substrates [7,24,26,38].

Importantly, in our study we could show that our product **1c** (and all other products in this study) can be separated from the PVPy-IL-B-SO_3_H catalyst by simple filtration and the purification required only re-crystallization and not laborious column chromatography as in all previous reports, even including those with easily removable catalysts [4,7,19,20,35,36,37,38,39].

### 2.3. Mechanistical Considerations

**Scheme 4:**
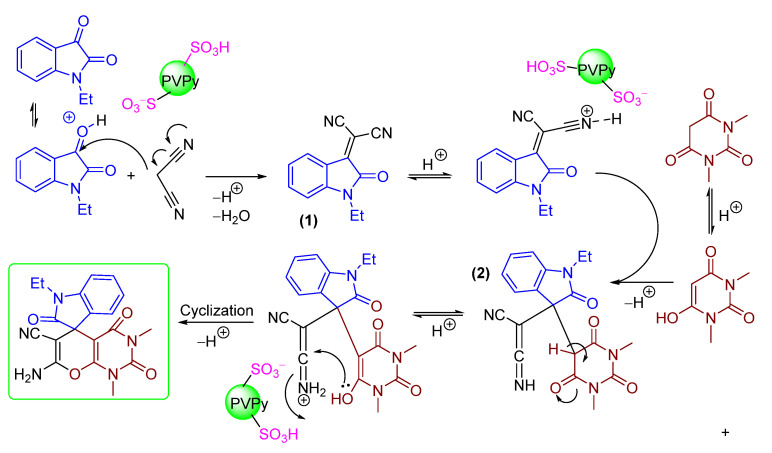
Proposed mechanism of the formation of the pyrimidine **1c**.

Mechanistically, we assume that the reaction is initiated by protonation of the 1-ethylindoline-2,3-dione at the carbonyl group enabling the nucleophilic attack of the malononitrile (Scheme 4).

A Knoevenagel-like condensation then leads to the formation of the intermediate **1**. Similar intermediates have previously been isolated and characterized, underpinning our assumption [5,7,13,24]. The reaction continues with the nucleophilic attack of the enolic form of 1,3-dimethylpyrimidine-2,4,6(1*H*,3*H*,5*H*)-trione to the intermediate **1** similar to a Michael addition leading to the formation of the intermediate **2** [5,7,18,24]. The protonation of the imine nitrogen in **2** and the formation of an enolic structure in the pyrimidine core provides the conditions for the cyclization reaction, which leads to the product **1c**. This proposed mechanism is completely in line with mechanistic studies on similar spirooxindole products [5,7,13,18,24].

We assume that the pyridinium/SO_3_H^−^ ionic liquid moiety is slightly more acidic than the sulfonic acid chain, but both very probably contribute to the protonation-deprotonation reactions in the catalytic cycle.

### 2.4. Synthesis of Spiro-Indoline-3,5′-pyrano[2,3-*d*]pyrimidine Derivatives—Substrate Scope

With the optimized conditions (Table 1) in hand, other substrates were studied (Table 2). In general, all reactions produced excellent yields (89–97%). Replacing 1,3-dimethylpyrimidine-2,4,6(1*H*,3*H*,5*H*)-trione by the bulkier 1,3-dibenzylpyrimidine-2,4,6(1*H*,3*H*,5*H*)-trione (Table 2, product **3c**) and introduction of phenylethyl to the dione (product **6c**) required slightly extended reaction times to obtain full conversion and high yields.

In a recent study, isatin was reacted with malonitrile and 1,3-dimethylpyrimidine-2,4,6(1*H*,3*H*,5*H*)-trione with oleic acid as homogeneous acid catalyst and the NH (instead of NEt) derivative of **1c** was obtained in 89% yield [22]. For the corresponding pyrimidine-2,4,6(1*H*,3*H*,5*H*)-trione (2 × NH on the pyrimidine), a slightly increased yield of 91% was recorded [22], in line with our findings that the N-substitution on the pyrimidines has no impact on the yield. More interesting is the drop of the yield to 73% when using the malonic cyanide ethyl ester NC–CH_2_–COOEt instead of malonitrile [22]. Such a decrease in yield was not observed for the analogous reactions with 1,3-diketones using the so-called Carbon-SO_3_H catalyst [20]. We assume that the higher acidity of the pyridinium/HSO_3_^−^ and the butane–SO_3_H functions compared with oleic acid overcomes the lower reactivity of the ester in line with our mechanistic considerations on the protonation of intermediate **2** (Scheme 4).

Interestingly, the previously reported four-component reactions of 1-alkylindoline-2,3-diones, and 1,3-dimethylpyrimidine-2,4,6(1*H*,3*H*,5*H*)-trione with alkylamines and nitroketene dithioacetals allowed us to synthesize a number of spiro-indoline-3,5′-pyrano[2,3-*d*]pyrimidines with R_1_ = Me, Et, *i*-Pr, Bn and R = H, Me, or Bn comparable to our series [12,15].

### 2.5. Synthesis of Spiro-Indoline-3,5′-pyrano[2,3-*d*]pyrazole Derivatives—Substrate Scope

The optimized conditions were applied for the three-component reaction of substituted indoline-2,3-diones, malononitrile and methyl 5-hydroxy-1*H*-pyrazole-3-carboxylate leading to the formation of the pyrazoles **1d**–**8d** (Table 3). Reaction times between 60 to 90 min allowed for obtaining full conversion and high yields of 89 to 97%. They include 1-alkylindoline-2,3-diones with electron-withdrawing (X = 5-Cl, 5,7-Cl_2_, 5-Br, and 5,7-Br_2_) or electron-donating (X = 5-Me, 5-OMe, 5,7-Me_2_) substituents on the phenyl group and various alkyl groups on the indole N atom (R = Et, *n*-Pr, Bn, EtPh).

In a similar study, Mn_2_O_3_ nanoparticles were used as catalysts, and a similar pyrazole with R = H and a CH_3_ substituent instead of the ester function was produced in 97% yield [33]. Using the Carbon-SO_3_H catalyst, the yield for this reaction was 90% [20]. The NPh derivative was obtained in 86% using SiO_2_-SO_3_H as catalyst [19].

In a comprehensive study similar to ours, substituents were varied on the isatin phenyl core when synthesizing NPh (pyrazine) derivatives [23]. As in our study, the introduction of substituents ranging from electron-donating CH_3_ to electron-withdrawing Br or NO_2_ did not alter the yields markedly [23]. The same was found for H, Cl, Br or NO_2_ substituted isatins [20]. This study did also not reveal any difference between NH or NPh derivatives on the pyrazine concerning the yields.

**Figure 4:**
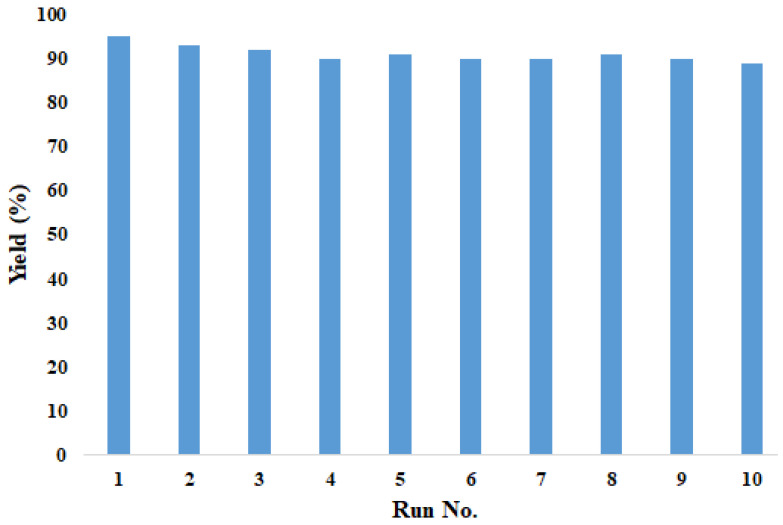
Yields of pyrazole **1d** using recovered catalyst in 10 subsequent runs. Conditions as in Table 3.

Finally, we studied the synthesis of the pyrazole **1d** using a recovered catalyst (Figure 4). In 10 subsequent runs, the yields decreased from 95 to about 90%.

In future work, we will study the reuse and recycling of the PVPy-IL-B-SO_3_H catalyst in more detail, as well as its biodegradability.

## 3. Materials and Methods

### 3.1. Syntheses

#### 3.1.1. Preparation of PVP IL-B-SO_3_H

We added 5.45 g (4.1 mL, 40 mmol) 1,2-oxathiane-2,2-dioxide dropwise to a solution of 4.21 g (40 mmol) 4-vinylpyridine in THF (100 mL) placed in a 250 mL flask equipped with a condenser. The mixture was stirred at 90 °C for 5 h under reflux. After completion of the reaction, the mixture was acidified using conc. HCl and stirred overnight. Next, THF was distilled off and the resulting 1-(4-sulfobutyl)-4-vinylpyridin-1-ium chloride was dried in vacuum. Then, 8.33 g (30 mmol) 1-(4-sulfobutyl)-4-vinylpyridin-1-ium chloride was polymerized in a pressure vessel using 493 mg (3 mmol) azobisisobutyronitrile (AIBN) in 50 mL toluene at 100 °C for 5 h. For the purification of the polymer, the solvent was evaporated under reduced pressure and the remaining solid was washed twice with 20 mL Et_2_O. After drying, the solid was weighted and the monomer conversion determined to 63%. Finally, 4 g (40 mmol) H_2_SO_4_ (98%) was added to the mixture, stirred for 5 h at 50 °C and subsequently filtered, washed with diethyl ether (3 × 20 mL) and dried. Elemental analysis produced C, 39.25; H, 5.49; N, 4.04; S, 17.98%. Calculated values for complete chain sulfonation, pyridine-alkylation, and Cl^−^ to ^−^SO_3_H replacement (C_11_H_17_NO_7_S_2_, M_W_ = 339.38 g/mol, see Scheme 3) were C 38.93; H, 5.05; N, 4.13; O, 33.00; and S, 18.89%.

#### 3.1.2. Acidity Measurement Using the Barium Sulfate Test

1 g of the as-prepared catalyst was dispersed in 100 mL of deionized water and combined with a solution of H_2_O_2_ (50 mL, 30%) and NaOH (2 g), and the resulting mixture was stirred for 2 h at 50 °C to convert all sulfonic groups to sulfate (SO_4_^2−^) ions. Next, the solution was titrated with a solution of BaCl_2_ (1 M). The precipitated BaSO_4_ was collected, dried and carefully weighed. The amount of sulfate ions accounts for 5.06 mmol/g catalyst. Accordingly, the total H^+^ capacity of the catalyst was 5.06 mmol H^+^/g.

#### 3.1.3. General Procedure for the Preparation of the Pyrimidines (**1**–**6**)**c**

In typical reaction in a 25 mL flask equipped with a condenser, 0.04 g of PVP IL-B-SO_3_H was added to a mixture of 66.1 mg (1 mmol) malononitrile, 175 mg (1 mmol) 1-ethylindoline-2,3-dione, and 260 mg (1 mmol) 1,3-dimethylpyrimidine-2,4,6(1*H*,3*H*,5*H*)-trione in 10 mL EtOH under stirring. The mixture was irradiated in an ultrasonic bath at 80 °C for 50 min. The progress of the reaction was monitored by thin-layer chromatography (TLC). After completion, the catalyst was filtered off using a paper filter, washed with acetone and dried. The filtrate was evaporated to dryness and the crude product was purified by recrystallization from EtOH.

7′-amino-1-ethyl-1′,3′-dimethyl-2,2′,4′-trioxo-1′,2′,3′,4′-tetrahydrospiro[indoline-3,5′-pyrano[2,3-*d*]pyrimidine]-6′-carbonitrile (Table 2, 1c): Colorless powder, m.p.: 289–291 °C; ^1^H NMR (500 MHz, DMSO-d_6_): δ = 1.18 (t, *J* = 7.3 Hz, 3H), 3.11 (s, 3H), 3.49 (s, 3H), 3.84 (q, *J* = 7.3 Hz, 2H), 6.94 (d, *J* = 7.8 Hz, 1H), 7.15 (t, *J* = 7.8 Hz, 1H), 7.28 (s, 2H), 7.29 (d, *J* = 7.8 Hz, 1H), 7.48 (t, *J* = 7.8 Hz, 1H) ppm; ^13^C NMR (125 MHz, DMSO-d_6_): δ = 13.7, 48.7, 49.7, 50.6, 51.4, 58.2, 87.4, 110.5, 118.2, 122.0, 124.5, 129.3, 134.1, 142.8, 150.7, 154.4, 160.5, 163.3, 177.1 ppm; Found: C, 60.26; H, 4.58; N, 18.41% C_19_H_17_N_5_O_4_; Requires: C, 60.15; H, 4.52; N, 18.46%.

7′-amino-1,1′,3′-triethyl-2,2′,4′-trioxo-1′,2′,3′,4′-tetrahydrospiro[indoline-3,5′-pyrano[2,3-*d*]pyrimidine]-6′-carbonitrile (Table 2, 2c): Colorless powder, m.p.: 285–257 °C; ^1^H NMR (500 MHz, DMSO-d_6_): δ = 1.17–1.24 (m, 9H), 3.19 (q, *J* = 7.2 Hz, 2H), 3.56 (q, *J* = 7.2 Hz, 2H), 3.82 (q, *J* = 7.4 Hz, 2H), 6.96 (d, *J* = 8.0 Hz, 1H), 7.16 (t, *J* = 8.0 Hz, 1H), 7.42 (s, 2H), 7.28 (d, *J* = 8.0 Hz, 1H), 7.50 (t, *J* = 8.0 Hz, 1H) ppm; ^13^C NMR (125 MHz, DMSO-d_6_): δ = 12.8, 13.2, 13.7, 47.5, 48.9, 50.4, 51.2, 58.4, 87.9, 110.2, 118.0, 122.6, 124.7, 129.2, 134.4, 143.1, 151.1, 154.8, 159.4, 162.3, 177.9 ppm; Found: C, 62.02; H, 5.34; N, 17.13% C_21_H_21_N_5_O_4_; Requires: C, 61.91; H, 5.20; N, 17.19%.

7′-amino-1′,3′-dibenzyl-1-ethyl-2,2′,4′-trioxo-1′,2′,3′,4′-tetrahydrospiro[indoline-3,5′-pyrano[2,3-*d*]pyrimidine]-6′-carbonitrile (Table 2, 3c): Colorless powder, m.p.: >300 °C; ^1^H NMR (500 MHz, DMSO-d_6_): δ = 1.21 (t, *J* = 7.2 Hz, 2H), 3.83 (q, *J* = 7.2 Hz, 2H), 4.19 (d, *J* = 11.2 Hz, 1H), 4.26 (d, *J* = 11.2 Hz, 1H), 4.49 (d, *J* = 11.0 Hz, 1H), 4.56 (d, *J* = 11.0 Hz, 1H), 6.95 (d, *J* = 8.4 Hz, 1H), 7.14–7.35 (m, 12H), 7.52 (t, *J* = 8.4 Hz, 1H), 7.72 (s, 2H) ppm; ^13^C NMR (125 MHz, DMSO-d_6_): δ = 13.8, 50.3, 51.4, 58.2, 74.6, 77.5, 88.6, 110.2, 118.2, 122.4, 124.5, 127.2, 127.4, 128.0, 128.4, 128.6, 128.9, 129.6, 135.6, 137.4, 138.1, 143.0, 151.4, 155.2, 159.7, 162.4, 179.1 ppm; Found: C, 69.95; H, 4.66; N, 13.27% C_31_H_25_N_5_O_4_; Requires: C, 70.05; H, 4.74; N, 13.18%.

7′-amino-1′,3′-dimethyl-2,2′,4′-trioxo-1-propyl-1′,2′,3′,4′-tetrahydrospiro[indoline-3,5′-pyrano[2,3-*d*]pyrimidine]-6′-carbonitrile (Table 2, 4c): Colorless powder, m.p.: 278–280 °C; ^1^H NMR (500 MHz, DMSO-d_6_): δ = 0.98 (t, *J* = 7.3 Hz, 3H), 1.21 (t, *J* = 7.4 Hz, 3H), 1.20 (q, *J* = 7.2 Hz, 2H), 3.15 (s, 3H), 3.49 (s, 3H), 3.82 (t, *J* = 7.4 Hz, 2H), 6.95 (d, *J* = 8.0 Hz, 1H), 7.17 (t, *J* = 8.0 Hz, 1H), 7.29 (d, *J* = 8.0 Hz, 1H), 7.31 (s, 2H), 7.49 (t, *J* = 8.0 Hz, 1H) ppm; ^13^C NMR (125 MHz, DMSO-d_6_): *δ* = 11.8, 14.9, 48.6, 49.8, 50.6, 51.5, 58.6, 87.5, 110.3, 118.0, 122.1, 124.5, 129.0, 134.4, 142.9, 150.2, 154.6, 161.2, 163.6, 177.4 ppm; Found: C, 60.97; H, 4.79; N, 17.87% C_20_H_19_N_5_O_4_; Requires: C, 61.06; H, 4.87; N, 17.80%.

7′-amino-1-benzyl-1′,3′-dimethyl-2,2′,4′-trioxo-1′,2′,3′,4′-tetrahydrospiro[indoline-3,5′-pyrano[2,3-*d*]pyrimidine]-6′-carbonitrile (Table 2, 5c): Colorless powder, m.p.: >300 °C; ^1^H NMR (500 MHz, DMSO-d_6_): δ = 3.14 (s, 3H), 3.51 (s, 3H), 4.51 (d, *J* = 11.0 Hz, 1H), 4.62 (d, *J* = 11.0 Hz, 1H), 6.96 (d, *J* = 8.2 Hz, 1H), 7.16–7.41 (m, 9H), 7.51 (t, *J* = 8.2 Hz, 1H) ppm; ^13^C NMR (125 MHz, DMSO-d_6_): δ = 49.9, 50.7, 51.6, 58.6, 68.6, 87.4, 110.6, 117.9, 122.2, 124.5, 127.3, 128.1, 128.6, 129.2, 134.7, 137.6, 143.2, 150.2, 154.7, 161.2, 163.5, 178.8 ppm; Found: C, 65.41; H, 4.39; N, 15.83% C_24_H_19_N_5_O_4_; Requires: C, 65.30; H, 4.34; N, 15.86%.

7′-amino-1′,3′-dimethyl-2,2′,4′-trioxo-1-phenethyl-1′,2′,3′,4′-tetrahydrospiro[indoline-3,5′-pyrano[2,3-*d*]pyrimidine]-6′-carbonitrile (Table 2, 6c): Colorless powder, m.p.: >300 °C; ^1^H NMR (500 MHz, DMSO-d_6_): δ = 2.63 (t, *J* = 7.6 Hz, 2H), 3.14 (s, 3H), 3.49 (s, 3H), 4.93 (t, *J* = 7.6 Hz, 2H), 6.95 (d, *J* = 8.2 Hz, 1H), 7.04 (d, *J* = 7.8 Hz, 2H), 7.11–7.31 (m, 7H), 7.50 (t, *J* = 8.2 Hz, 1H) ppm; ^13^C NMR (125 MHz, DMSO-d_6_): δ = 23.1, 49.4, 50.2, 51.5, 58.4, 59.6, 87.4, 110.7, 118.2, 122.1, 124.6, 127.1, 128.0, 128.7, 129.4, 134.7, 136.7, 143.1, 150.2, 154.5, 161.2, 163.3, 177.6 ppm; Found: C, 65.95; H, 4.72; N, 15.31% C_25_H_21_N_5_O_4_; Requires: C, 65.93; H, 4.65; N, 15.38%.

#### 3.1.4. General Procedure for the Preparation of (**1**–**8**)**d**

In a typical reaction, a 25 mL flask was equipped with a condenser, and 40 mg of PVPy-IL-B-SO_3_H was added to a mixture of 66.1 mg (1 mmol) malononitrile, 175 mg (1 mmol) indoline-2,3-dione, and 156 g (1 mmol) methyl 5-hydroxy-1*H*-pyrazole-3-carboxylate, in 10 mL EtOH under stirring. The mixture was irradiated in an ultrasonic bath at 80 °C for 50 min. The progress of the reaction was monitored by thin-layer chromatography (TLC). After completion, the catalyst was filtered off using a paper filter, washed with acetone and dried. The filtrate was evaporated to dryness and the crude product was purified by recrystallization from EtOH.

6′-amino-5′-cyano-5-methyl-2-oxo-2′*H*-spiro[indoline-3,4′-pyrano[2,3-*c*]pyrazole]-3′-carboxylate (Table 3, 1d): Colorless powder, m.p.: 269–271 °C; ^1^H NMR (500 MHz, DMSO-d_6_): δ = 11.41 (s, 1H), 7.12 (s, 2H), 7.02 (d, *J* = 8.3 Hz, 1H), 6.97 (s, 1H), 6.85 (s, 1H), 6.73 (d, *J* = 8.3 Hz, 1H), 3.51 (s, 3H), 2.37 (s, 3H) ppm; ^13^C NMR (125 MHz, DMSO-d_6_): δ = 180.3, 164.6, 162.3, 159.4, 156.4, 142.3, 136.7, 130.5, 127.4, 124.5, 118.7, 111.9, 103.3, 57.4, 52.6, 48.8, 21.4 ppm; Found: C, 58.03; H, 3.68; N, 19.85% C_17_H_13_N_5_O_4_; Requires: C, 58.12; H, 3.73; N, 19.93%.

6′-amino-5′-cyano-5-methoxy-2-oxo-2′*H*-spiro[indoline-3,4′-pyrano[2,3-*c*]pyrazole]-3′-methyl ester (Table 3, 2d): Colorless powder, m.p.: 273–275 °C; ^1^H NMR (500 MHz, DMSO-d_6_): δ = 10.12 (s, 1H), 6.91 (d, *J* = 8.4 Hz, 1H), 6.86 (s, 1H), 6.78 (d, *J* = 8.4 Hz, 1H), 6.74 (s, 1H), 6.73 (s, 2H), 3.73 (s, 3H), 3.55 (s, 3H) ppm; ^13^C NMR (125 MHz, DMSO-d_6_): δ = 181.4, 164.7, 162.1, 159.0, 156.7, 154.8, 142.4, 129.9, 126.7, 124.0, 117.7, 107.5, 103.4, 57.5, 55.7, 52.5, 48.9 ppm; Found: C, 55.53; H, 3.51; N, 19.14% C_17_H_13_N_5_O_5_; Requires: C, 55.59; H, 3.57; N, 19.07%.

6′-amino-5-chloro-5′-cyano-2-oxo-2′*H*-spiro[indoline-3,4′-pyrano[2,3-*c*]pyrazole]-3′-methyl ester (Table 3, 3d): Colorless powder, m.p.: 286–288 °C; ^1^H NMR (500 MHz, DMSO-d_6_): δ = 10.97 (s, 1H), 7.26 (d, *J* = 8.1 Hz, 1H), 7.19 (s, 2H), 7.14 (s, 1H), 6.98 (d, *J* = 8.3 Hz, 1H), 6.95 (s, 1H), 3.52 (s, 3H), ppm; ^13^C NMR (125 MHz, DMSO-d_6_): δ = 183.4, 164.9, 162.1, 158.7, 156.5, 142.6, 140.5, 129.7, 128.7, 126.7, 124.7, 112.5, 103.6, 57.5, 52.6, 49.2 ppm; Found: C, 51.76; H, 2.79; N, 18.88% C_16_H_10_ClN_5_O_4_; Requires: C, 51.70; H, 2.71; N, 18.84%.

6′-amino-5-bromo-5′-cyano-2-oxo-2′*H*-spiro[indoline-3,4′-pyrano[2,3-*c*]pyrazole]-3′-methyl ester (Table 3, 4d): Colorless powder, m.p.: >300 °C; ^1^H NMR (500 MHz, DMSO-d_6_): δ = 10.24 (s, 1H), 7.33 (d, *J* = 8.1 Hz, 1H), 7.21 (s, 1H), 7.19 (s, 2H), 7.13 (d, *J* = 8.1 Hz, 1H), 6.94 (s, 1H), 3.53 (s, 3H) ppm; ^13^C NMR (125 MHz, DMSO-d_6_): δ = 183.1, 164.8, 162.1, 158.7, 156.4, 147.5, 142.5, 130.7, 129.7, 127.7, 124.9, 114.5, 104.1, 57.7, 52.8, 50.2 ppm; Found: C, 46.23; H, 2.49; N, 16.94% C_16_H_10_BrN_5_O_4_; Requires: C, 46.17; H, 2.42; N, 16.83%.

6′-amino-5′-cyano-5,7-dimethyl-2-oxo-2′*H*-spiro[indoline-3,4′-pyrano[2,3-*c*]pyrazole]-3′-methyl ester (Table 3, 5d): Colorless powder, m.p.: >300 °C; ^1^H NMR (500 MHz, DMSO-d_6_): δ = 10.23 (s, 1H), 7.09 (s, 2H), 6.89 (s, 1H), 6.74 (s, 1H), 6.69 (s, 1H), 3.53 (s, 3H), 2.14 (s, 3H), 2.23 (s, 3H) ppm; ^13^C NMR (125 MHz, DMSO-d_6_): δ = 179.1, 163.8, 162.4, 158.9, 154.3, 140.1, 136.1, 135.2, 124.4, 119.3, 117.7, 110.7, 103.2, 57.7, 52.6, 48.9, 21.4, 20.3 ppm; Found: C, 59.27; H, 4.25; N, 19.08% C_18_H_15_N_5_O_4_; Requires: C, 59.18; H, 4.14; N, 19.17%.

6′-amino-5,7-dibromo-5′-cyano-2-oxo-2′*H*-spiro[indoline-3,4′-pyrano[2,3-*c*]pyrazole]-3′-methyl ester (Table 3, 6d): Colorless powder, m.p.: >300 °C; ^1^H NMR (500 MHz, DMSO-d_6_): δ = 11.023 (s, 1H), 7.28 (s, 2H), 6.91 (s, 1H), 7.47 (s, 1H), 7.64 (s, 1H), 3.53 (s, 3H) ppm; ^13^C NMR (125 MHz, DMSO-d_6_): δ = 182.1, 164.2, 163.1, 158.9, 154.5, 148.1, 146.1, 140.4, 130.4, 129.3, 118.7, 111.4, 103.6, 57.9, 53.4, 50.1 ppm; Found: C, 38.74; H, 1.80; N, 14.07% C_16_H_9_Br_2_N_5_O_4_; Requires: C, 38.82; H, 1.83; N, 14.15%.

6′-amino-5,7-dichloro-5′-cyano-2-oxo-2′*H*-spiro[indoline-3,4′-pyrano[2,3-*c*]pyrazole]-3′-methyl ester (Table 3, 7d): Colorless powder, m.p.: >300 °C; ^1^H NMR (500 MHz, DMSO-d_6_): δ = 10.70 (s, 1H), 7.27 (s, 1H), 7.16 (s, 2H), 7.04 (s, 1H), 6.71 (s, 1H), 3.51 (s, 3H) ppm; ^13^C NMR (125 MHz, DMSO-d_6_): δ = 180.8, 163.8, 162.7, 158.7, 154.8, 142.4, 140.7, 140.1, 133.1, 130.7, 118.9, 111.1, 103.4, 57.8, 53.1, 49.6 ppm; Found: C, 47.43; H, 2.31; N, 17.21% C_16_H_9_Cl_2_N_5_O_4_; Requires: C, 47.31; H, 2.23; N, 17.24%.

6′-amino-5,7-dichloro-5′-cyano-2-oxo-1-phenyl-2′*H*-spiro[indoline-3,4′-pyrano[2,3-*c*]pyrazole]-3′-methyl ester (Table 3, 8d): Colorless powder, m.p.: >300 °C; ^1^H NMR (500 MHz, DMSO-d_6_): δ = 7.61 (d, *J* = 8.0 Hz, 2H), 7.52 (t, *J* = 8.1 Hz, 1H), 7.39 (t, *J* = 8.0 Hz, 2H), 7.28 (s, 2H), 7.27 (s, 1H), 6.78 (s, 1H), 7.09 (s, 1H), 3.52 (s, 3H) ppm; ^13^C NMR (125 MHz, DMSO-d_6_): δ = 181.7, 169.1, 163.1, 158.6, 154.9, 142.4, 140.9, 140.2, 139.4, 133.1, 130.9, 126.3, 121.3, 119.2, 118.8, 110.5, 103.1, 57.6, 52.8, 49.8 ppm; Found: C, 54.85; H, 2.77; N, 14.45% C_22_H_13_Cl_2_N_5_O_4_; Requires: C, 54.79; H, 2.72; N, 14.52%.

#### 3.1.5. Catalyst Recovery and Re-Use Tests

For the repeated synthesis of the pyrazole 1d, the catalyst was recovered from the reaction by filtration after each run, washed with EtOH and dried. Thus, 10 subsequent reaction cycles were carried out.

### 3.2. Instrumentation

NMR spectra were recorded on a Bruker Avance DPX 500 MHz instrument in DMSO-d_6_ (Bruker, Rheinhausen, Germany). Chemical shifts were provided relative to TMS. Elemental analysis was measured using a Heraeus CHN-O-Rapid analyzer (Heraeus, Hanau, Germany). Fourier-transformed IR (FT-IR) was carried out on a KBr pellet using a Shimadzu FT-IR-8400 instrument (Shimadzu, Duisburg, Germany). TGA-DTA was measured using a PL Thermal Science STA-1500 Polymer Thermal Analyser (PL Thermal Science, Boxhill, UK). Field emission scanning electron microscopy (FE-SEM) was carried out on FEI NOVA NANOSEM 450 (Thermo Fisher Scientific, Waltham, MA, USA).

## 4. Conclusions

A new sulfonic acid functionalized polymeric acid catalyst PVPy-IL-B-SO_3_H containing a pyridinium/HSO_3_^−^ ionic liquid moiety was obtained from the reaction of 4-vinyl pyridine with 1,2-oxathiane 2,2-dioxide and polymerization of the resulting 4-(4-vinylpyridin-1-ium-1-yl)butane-1-sulfonate and subsequent treatment with H_2_SO_4_. Elemental analysis showed about 5 mmol sulfate groups per gram catalyst. Using 0.04 g (~0.2 mmol) PVPy-IL-B-SO_3_H, we catalyzed the formation of a number of functionalized spirooxindoles (**1c**–**6c** and **1d**–**8d**) in the (1:1:1) three-component reaction of malononitrile with 1-alkylindoline-2,3-dione and 1,3-dimethylpyrimidine-2,4,6(1*H*,3*H*,5*H*)-trione or methyl 5-hydroxy-1*H*-pyrazole-3-carboxylate under ultrasonic irradiation (US). High isolated yields ranging from 89 to 97% were obtained in refluxing EtOH within 50 to 90 min for various substrates, including bulky 1,3-dialkylpyrimidine-2,4,6(1*H*,3*H*,5*H*)-triones (R_1_ = Me, Et, Bn) for the synthesis of pyrimidines and 1-alkylindoline-2,3-diones with electron-withdrawing (5-X, 5,7-X_2_, X = Cl or Br) or electron-donating (5-Me, 5-OMe, 5,7-Me_2_) substituents on the phenyl group and various alkyl groups on the indole N atom (Et, *n*-Pr, Bn, EtPh) for the synthesis of pyrazine derivatives. The catalyst was easily separable, and product isolation did not require column chromatography for isolation and purification. Catalyst recovery experiments showed only a slight decrease in yield from 95 to about 90% within 10 runs of producing the pyrazole **1d**. The simple operation conditions and use of a cheap, metal-free and easy recoverable catalyst make our method advantageous over comparable procedures to synthesize such functionalized spirooxindoles. In future work, we will explore further reactions and substrates using PVPy-IL-B-SO_3_H as an acid catalyst as well as the re-usability and/or recyclability of the catalyst.

## Figures and Tables

**Table 1:** Optimization of the reaction conditions for compound **1c** ^a^.

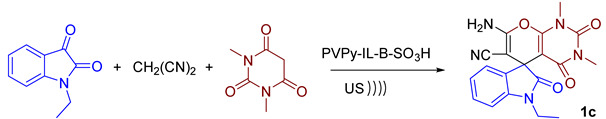				
**Entry**	**Catalyst (g) ^b^**	**Conditions ^c^**	**Time (min)**	**Yield (%) ^d^**
1	-	US; EtOH; reflux	70	-
2	0.04	US; EtOH; r.t.	70	-
3	0.04	US; EtOH; 50 ℃	50	55
4	0.04	US; EtOH; reflux	50	95
5	0.02	US; EtOH; reflux	70	53
6	0.06	US; EtOH; reflux	45	94
7	0.04	US; H_2_O; reflux	60	45
8	0.04	US; CH_3_CN; reflux	60	48
9	0.04	US; Et_2_O; reflux	70	-
10	0.04	US; *n*-hexane; reflux	70	-
11	0.04	US; EtOAc; reflux	60	-
12	0.04	US; toluene; 80 °C	60	-
13	0.04	US; DMF; 80 °C	60	23
14	0.04	US; CH_2_Cl_2_; reflux	70	-

^a^ Reactants were used in a 1:1:1 molar ratio; ^b^ 0.01 g of catalyst corresponds to about 0.05 mmol ^c^ US = ultrasonic irradiation. ^d^ Isolated yields. Marked in grey are the optimum conditions.

**Table 2:** Preparation of the pyrimidines **1c**–**6c** under optimized conditions ^a^.

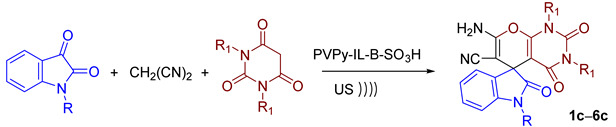					
**Product**	**R**	**R_1_**	**Time (min)**	**Yield (%) ^b^**	**M.p. (°C)**
**1c**	CH_3_CH_2_	CH_3_	50	95	289–291
**2c**	CH_3_CH_2_	CH_3_CH_2_	50	94	285–287
**3c**	CH_3_CH_2_	Ph–CH_2_	60	93	˃300
**4c**	CH_3_CH_2_CH_2_	CH_3_	50	89	278–280
**5c**	Ph–CH_2_	CH_3_	50	93	˃300
**6c**	Ph–CH_2_CH_2_	CH_3_	60	97	˃300

^a^ Conditions: 0.04 g (~0.2 mmol) catalyst, 1 mmol of each substrate, EtOH, reflux, ultrasound. ^b^ Isolated yields at complete conversion (TLC). The ^1^H and ^13^C NMR spectra of the products are shown in Figures S3–S12 in the Supplementary Material.

**Table 3:** Preparation of the pyrazoles **1d**–**8d** under optimized conditions ^a^.

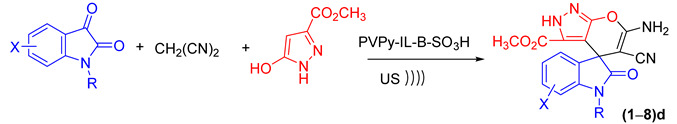					
**Product**	**R**	**X**	**Time (min)**	**Yield (%) ^b^**	**M.p. (°C)**
**1d**	H	5-CH_3_	90	95	269–271
**2d**	H	5-OCH_3_	90	93	273–275
**3d**	H	5-Cl	70	96	286–288
**4d**	H	5-Br	80	94	˃300
**5d**	H	5,7-CH_3_	90	95	˃300
**6d**	H	5,7-Br	60	97	˃300
**7d**	H	5,7-Cl	80	95	˃300
**8d**	Ph	5,7-Cl	75	89	˃300

^a^ Conditions: 0.04 g (~0.2 mmol) catalyst, 1 mmol of each substrate, EtOH, reflux, ultrasound. ^b^ Isolated yields at complete conversion (TLC). The ^1^H and ^13^C NMR spectra of the products are shown in Figures S13–S28 in the Supplementary Material.

## Data Availability

Available from the authors on request.

## Supplementary Materials

The following supporting information can be downloaded downloaded at: https://www.mdpi.com/article/10.3390/molecules28093663/s1, Figure S1: ^1^H NMR spectrum of 7′-amino-1-ethyl-1′,3′-dimethyl-2,2′,4′-trioxo-1′,2′,3′,4′-tetrahydrospiro[indoline-3,5′-pyrano[2,3-*d*]pyrimidine]-6′-carbonitrile (**1c**) in DMSO-d_6_. Figure S2: ^13^C NMR spectrum of 7′-amino-1-ethyl-1′,3′-dimethyl-2,2′,4′-trioxo-1′,2′,3′,4′-tetrahydrospiro[indoline-3,5′-pyrano[2,3-*d*]pyrimidine]-6′-carbonitrile (**1c**) in DMSO-d_6_. Figure S3: ^1^H NMR spectrum of 7′-amino-1,1′,3′-triethyl-2,2′,4′-trioxo-1′,2′,3′,4′-tetrahydrospiro[indoline-3,5′-pyrano[2,3-*d*]pyrimidine]-6′-carbonitrile (**2c**) in DMSO-d_6_. Figure S4: ^13^C NMR spectrum of 7′-amino-1,1′,3′-triethyl-2,2′,4′-trioxo-1′,2′,3′,4′-tetrahydrospiro[indoline-3,5′-pyrano[2,3-*d*]pyrimidine]-6′-carbonitrile (**2c**) in DMSO-d_6_. Figure S5: ^1^H NMR spectrum of 7′-amino-1′,3′-dibenzyl-1-ethyl-2,2′,4′-trioxo-1′,2′,3′,4′-tetrahydrospiro[indoline-3,5′-pyrano[2,3-*d*]pyrimidine]-6′-carbonitrile (**3c**) in DMSO-d_6_. Figure S6: ^13^C NMR spectrum of 7′-amino-1′,3′-dibenzyl-1-ethyl-2,2′,4′-trioxo-1′,2′,3′,4′-tetrahydrospiro[indoline-3,5′-pyrano[2,3-*d*]pyrimidine]-6′-carbonitrile (**3c**) in DMSO-d_6_. Figure S7: ^1^H NMR spectrum of 7′-amino-1′,3′-dimethyl-2,2′,4′-trioxo-1-propyl-1′,2′,3′,4′-tetrahydrospiro[indoline-3,5′-pyrano[2,3-*d*]pyrimidine]-6′-carbonitrile (**4c**) in DMSO-d_6_. Figure S8: ^13^C NMR spectrum of 7′-amino-1′,3′-dimethyl-2,2′,4′-trioxo-1-propyl-1′,2′,3′,4′-tetrahydrospiro[indoline-3,5′-pyrano[2,3-*d*]pyrimidine]-6′-carbonitrile (4c) in DMSO-d_6_. Figure S9: ^1^H NMR spectrum of 7′-amino-1-benzyl-1′,3′-dimethyl-2,2′,4′-trioxo-1′,2′,3′,4′-tetrahydrospiro[indoline-3,5′-pyrano[2,3-*d*]pyrimidine]-6′-carbonitrile (**5c**) in DMSO-d_6_. Figure S10: ^13^C NMR spectrum of 7′-amino-1-benzyl-1′,3′-dimethyl-2,2′,4′-trioxo-1′,2′,3′,4′-tetrahydrospiro[indoline-3,5′-pyrano[2,3-*d*]pyrimidine]-6′-carbonitrile (**5c**) in DMSO-d_6_. Figure S11: ^1^H NMR spectrum of 7′-amino-1′,3′-dimethyl-2,2′,4′-trioxo-1-phenethyl-1′,2′,3′,4′-tetrahydrospiro[indoline-3,5′-pyrano[2,3-*d*]pyrimidine]-6′-carbonitrile (**6c**) in DMSO-d_6_. Figure S12: ^13^C NMR spectrum of 7′-amino-1′,3′-dimethyl-2,2′,4′-trioxo-1-phenethyl-1′,2′,3′,4′-tetrahydrospiro[indoline-3,5′-pyrano[2,3-*d*]pyrimidine]-6′-carbonitrile (**6c**) in DMSO-d_6_. Figure S13: ^1^H NMR spectrum of methyl 6′-amino-5′-cyano-5-methyl-2-oxo-2′H-spiro[indoline-3,4′-pyrano[2,3-*c*]pyrazole]-3′-carboxylate (**1d**) in DMSO-d_6_. Figure S14: ^13^C NMR spectrum of methyl 6′-amino-5′-cyano-5-methyl-2-oxo-2′H-spiro[indoline-3,4′-pyrano[2,3-*c*]pyrazole]-3′-carboxylate (1d) in DMSO-d_6_. Figure S15: ^1^H NMR spectrum of methyl 6′-amino-5′-cyano-5-methoxy-2-oxo-2′H-spiro[indoline-3,4′-pyrano[2,3-*c*]pyrazole]-3′-carboxylate (**2d**) in DMSO-d_6_. Figure S16: ^13^C NMR spectrum of methyl 6′-amino-5′-cyano-5-methoxy-2-oxo-2′*H*-spiro[indoline-3,4′-pyrano[2,3-*c*]pyrazole]-3′-carboxylate (**2d**) in DMSO-d_6_. Figure S17: ^1^H NMR spectrum of methyl 6′-amino-5-chloro-5′-cyano-2-oxo-2′*H*-spiro[indoline-3,4′-pyrano[2,3-*c*]pyrazole]-3′-carboxylate (**3d**) in DMSO-d_6_. Figure S18: ^13^C NMR spectrum of methyl 6′-amino-5-chloro-5′-cyano-2-oxo-2′*H*-spiro[indoline-3,4′-pyrano[2,3-*c*]pyrazole]-3′-carboxylate (**3d**) in DMSO-d_6_. Figure S19: ^1^H NMR spectrum of methyl 6′-amino-5-bromo-5′-cyano-2-oxo-2′*H*-spiro[indoline-3,4′-pyrano[2,3-*c*]pyrazole]-3′-carboxylate (**4d**) in DMSO-d_6_. Figure S20: ^13^C NMR spectrum of methyl 6′-amino-5-bromo-5′-cyano-2-oxo-2′*H*-spiro[indoline-3,4′-pyrano[2,3-*c*]pyrazole]-3′-carboxylate (**4d**) in DMSO-d_6_. Figure S21: ^1^H NMR spectrum of methyl 6′-amino-5′-cyano-5,7-dimethyl-2-oxo-2′*H*-spiro[indoline-3,4′-pyrano[2,3-*c*]pyrazole]-3′-carboxylate (5d) in DMSO-d_6_. Figure S22: ^13^C NMR spectrum of methyl 6′-amino-5′-cyano-5,7-dimethyl-2-oxo-2′*H*-spiro[indoline-3,4′-pyrano[2,3-*c*]pyrazole]-3′-carboxylate (**5d**) in DMSO-d_6_. Figure S23: ^1^H NMR spectrum of methyl 6′-amino-5,7-dibromo-5′-cyano-2-oxo-2′*H*-spiro[indoline-3,4′-pyrano[2,3-*c*]pyrazole]-3′-carboxylate (**6d**) in DMSO-d_6_. Figure S24: ^13^C NMR spectrum of methyl 6′-amino-5,7-dibromo-5′-cyano-2-oxo-2′*H*-spiro[indoline-3,4′-pyrano[2,3-*c*]pyrazole]-3′-carboxylate (**6d**) in DMSO-d_6_. Figure S25: ^1^H NMR spectrum of methyl 6′-amino-5,7-dichloro-5′-cyano-2-oxo-2′*H*-spiro[indoline-3,4′-pyrano[2,3-*c*]pyrazole]-3′-carboxylate (**7d**) in DMSO-d_6_. Figure S26: ^13^C NMR spectrum of methyl 6′-amino-5,7-dichloro-5′-cyano-2-oxo-2′*H*-spiro[indoline-3,4′-pyrano[2,3-*c*]pyrazole]-3′-carboxylate (**7d**) in DMSO-d_6_. Figure S27: ^1^H NMR spectrum of methyl 6′-amino-5,7-dichloro-5′-cyano-2-oxo-1-phenyl-2′*H*-spiro[indoline-3,4′-pyrano[2,3-*c*]pyrazole]-3′-carboxylate (**8d**) in DMSO-d_6_. Figure S28: ^13^C NMR spectrum of methyl 6′-amino-5,7-dichloro-5′-cyano-2-oxo-1-phenyl-2′*H*-spiro[indoline-3,4′-pyrano[2,3-*c*]pyrazole]-3′-carboxylate (**8d**) in DMSO-d_6_.

## Sample Availability

Samples of the compounds are available from the authors.
