# Supplementary material for: A Sulfonic Acid Polyvinyl Pyridinium Ionic Liquid Catalyzes the Multi-Component Synthesis of Spiro-indoline-3,5′-pyrano[2,3-d]-pyrimidines and -Pyrazines
Source: Molecules. 2023 Apr 23;28(9):3663. doi: 10.3390/molecules28093663 (PMC10180120; doi:10.3390/molecules28093663)
Supplement: Supplementary file 1 [file molecules-28-03663-s001.zip › molecules-2325074-supplementary.pdf]

Supplementary Material
for

A sulfonic acid polyvinyl pyridinium ionic liquid catalyzes the multi-component synthesis of spiro-indoline-3,5'-pyrano[2,3-*d*]-pyrimidines and -pyrazines

Mehdi Khalaj[1,*] Mahboubeh Taherkhani[2], Leo Payen[3], and Axel Klein[*,3,]

[1] Department of Chemistry, Buinzahra Branch, Islamic Azad University, Buinzahra, Iran;
[2] Department of Chemistry, Takestan Branch, Islamic Azad University, Takestan, Iran, Email: mahtaherkhani@yahoo.com, ORCID: 0000-0002-4539-5937 (M.T.)
[3] University of Cologne, Faculty of Mathematics and Natural Sciences, Department of Chemistry, Institute for Inorganic Chemistry, Greinstrasse 6, 50939 Köln, Germany. Email: lpayen@smail.uni-koeln.de, ORCID: 0000-0003-1291-9690 (L.P.)
* Correspondence: E-mail: khalaj_mehdi@yahoo.com (M.K.), ORCID: 0000-0002-5327-9266; Email: axel.klein@uni-koeln.de, ORCID: 0000-0003-0093-9619 (A.K.).

Contents:
Figure S1. ^{1}H NMR spectrum of **1c** in DMSO-d_6.
Figure S2. ^{13}C NMR spectrum of **1c** in DMSO-d_6.
Figure S3. ^{1}H NMR spectrum of **2c** in DMSO-d_6.
Figure S4. ^{13}C NMR spectrum of **2c** in DMSO-d_6.
Figure S5. ^{1}H NMR spectrum of **3c** in DMSO-d_6.
Figure S6. ^{13}C NMR spectrum of **3c** in DMSO-d_6.
Figure S7. ^{1}H NMR spectrum of **4c** in DMSO-d_6.
Figure S8. ^{13}C NMR spectrum of **4c** in DMSO-d_6.
Figure S9. ^{1}H NMR spectrum of **5c** in DMSO-d_6.
Figure S10. ^{13}C NMR spectrum of **5c** in DMSO-d_6.
Figure S11. ^{1}H NMR spectrum of **6c** in DMSO-d_6.
Figure S12. ^{13}C NMR spectrum of **6c** in DMSO-d_6.
Figure S13. ^{1}H NMR spectrum of **1d** in DMSO-d_6.
Figure S14. ^{13}C NMR spectrum of **1d** in DMSO-d_6.
Figure S15. ^{1}H NMR spectrum of **2d** in DMSO-d_6.
Figure S16. ^{13}C NMR spectrum of **2d** in DMSO-d_6.
Figure S17. ^{1}H NMR spectrum of **3d** in DMSO-d_6.
Figure S18. ^{13}C NMR spectrum of **3d** in DMSO-d_6.
Figure S19. ^{1}H NMR spectrum of **4d** in DMSO-d_6.
Figure S20. ^{13}C NMR spectrum of **4d** in DMSO-d_6.
Figure S21. ^{1}H NMR spectrum of **5d** in DMSO-d_6.
Figure S22. ^{13}C NMR spectrum of **5d** in DMSO-d_6.
Figure S23. ^{1}H NMR spectrum of **6d** in DMSO-d_6.
Figure S24. ^{13}C NMR spectrum of **6d** in DMSO-d_6.
Figure S25. ^{1}H NMR spectrum of **7d** in DMSO-d_6.
Figure S26. ^{13}C NMR spectrum of **7d** in DMSO-d_6.
Figure S27. ^{1}H NMR spectrum of **8d** in DMSO-d_6.
Figure S28. ^{13}C NMR spectrum of **8d** in DMSO-d_6.

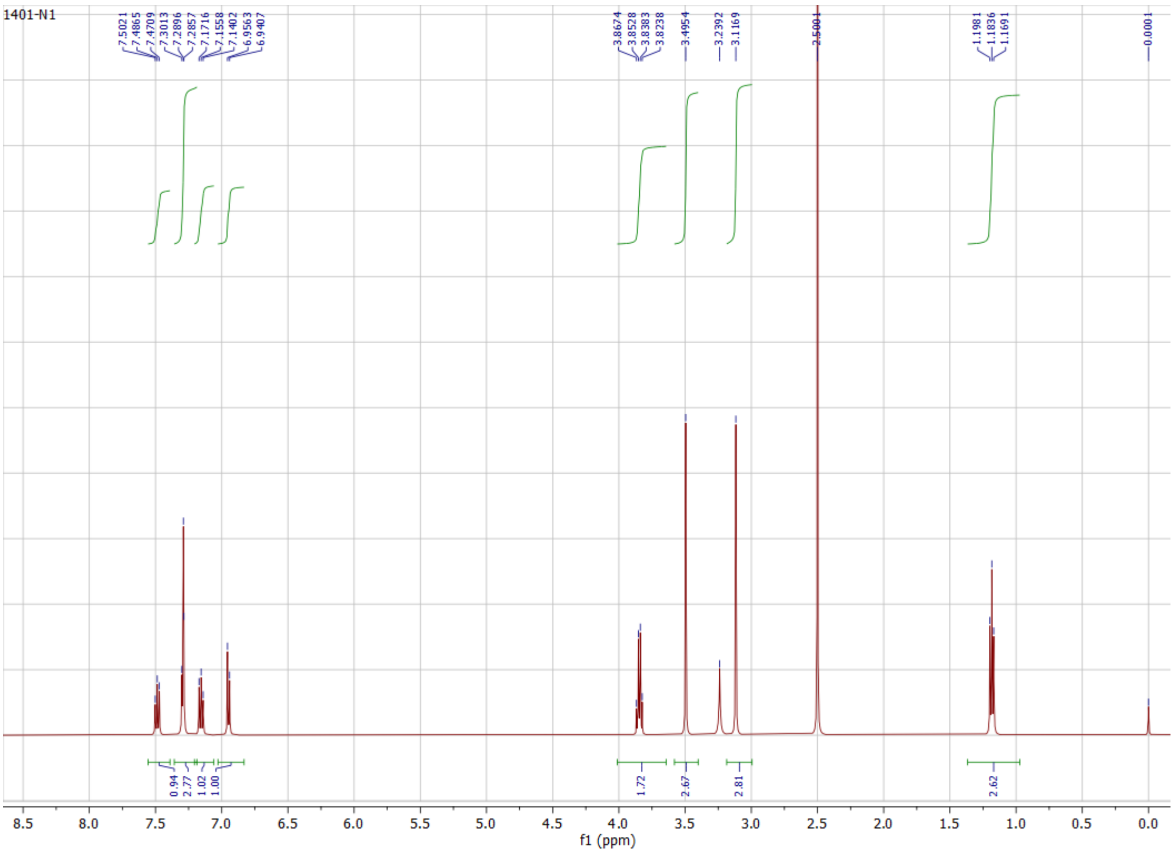

Figure S1. ^1H NMR spectrum of 7'-amino-1-ethyl-1',3'-dimethyl-2,2',4'-trioxo-1',2',3',4'-tetrahydrospiro[indoline-3,5'-pyrano[2,3-*d*]pyrimidine]-6'-carbonitrile (**1c**) in DMSO-d_6.

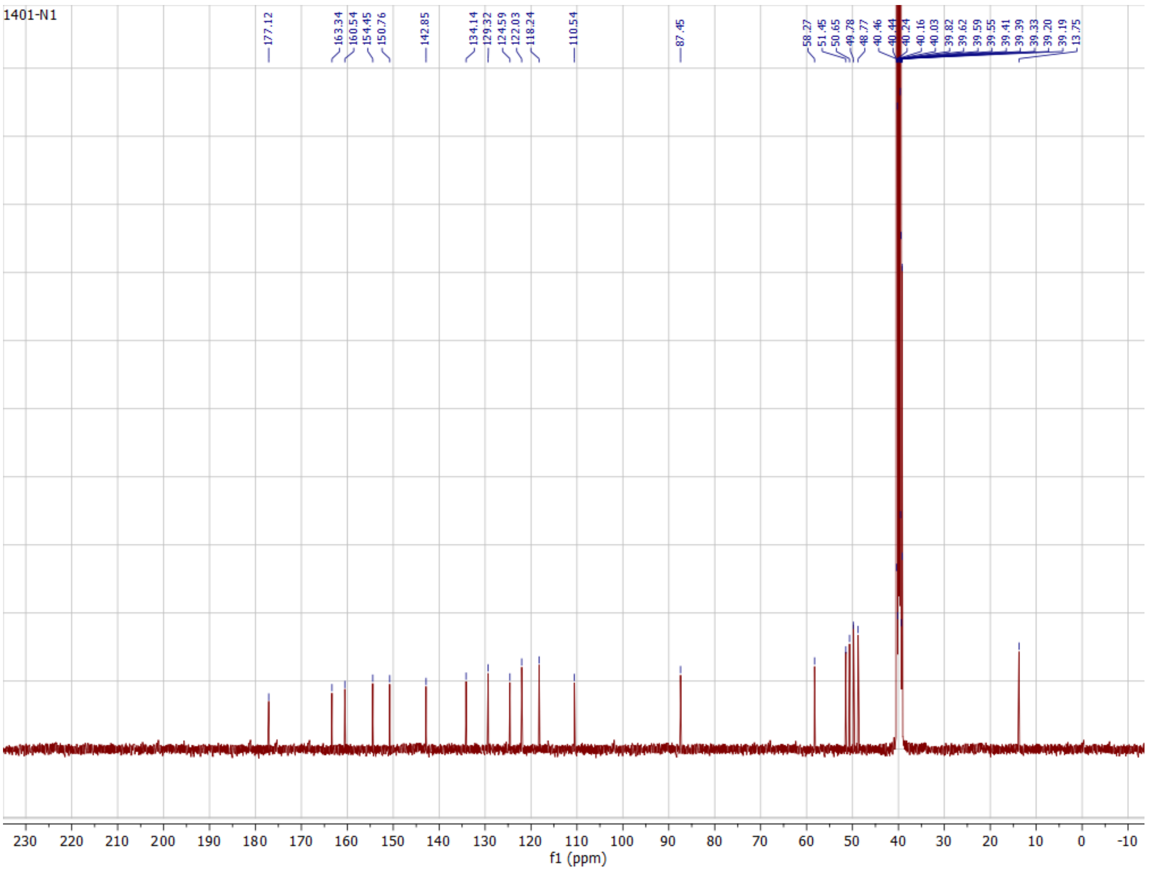

Figure S2. ^{13}C NMR spectrum of 7'-amino-1-ethyl-1',3'-dimethyl-2,2',4'-trioxo-1',2',3',4'-tetrahydrospiro[indoline-3,5'-pyrano[2,3-*d*]pyrimidine]-6'-carbonitrile (**1c**) in DMSO-d_6.

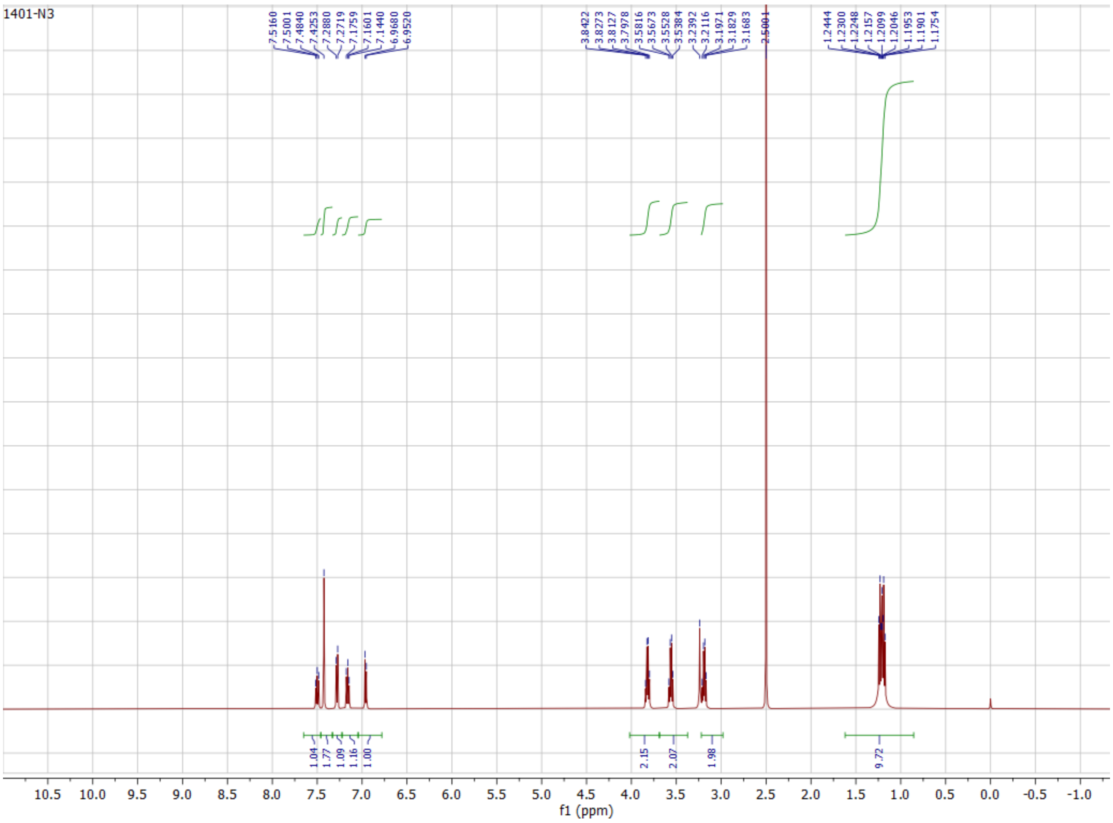

Figure S3. ^{1}H NMR spectrum of 7'-amino-1,1',3'-triethyl-2,2',4'-trioxo-1',2',3',4'-tetrahydrospiro[indoline-3,5'-pyrano[2,3-*d*]pyrimidine]-6'-carbonitrile (**2c**) in DMSO-d6.

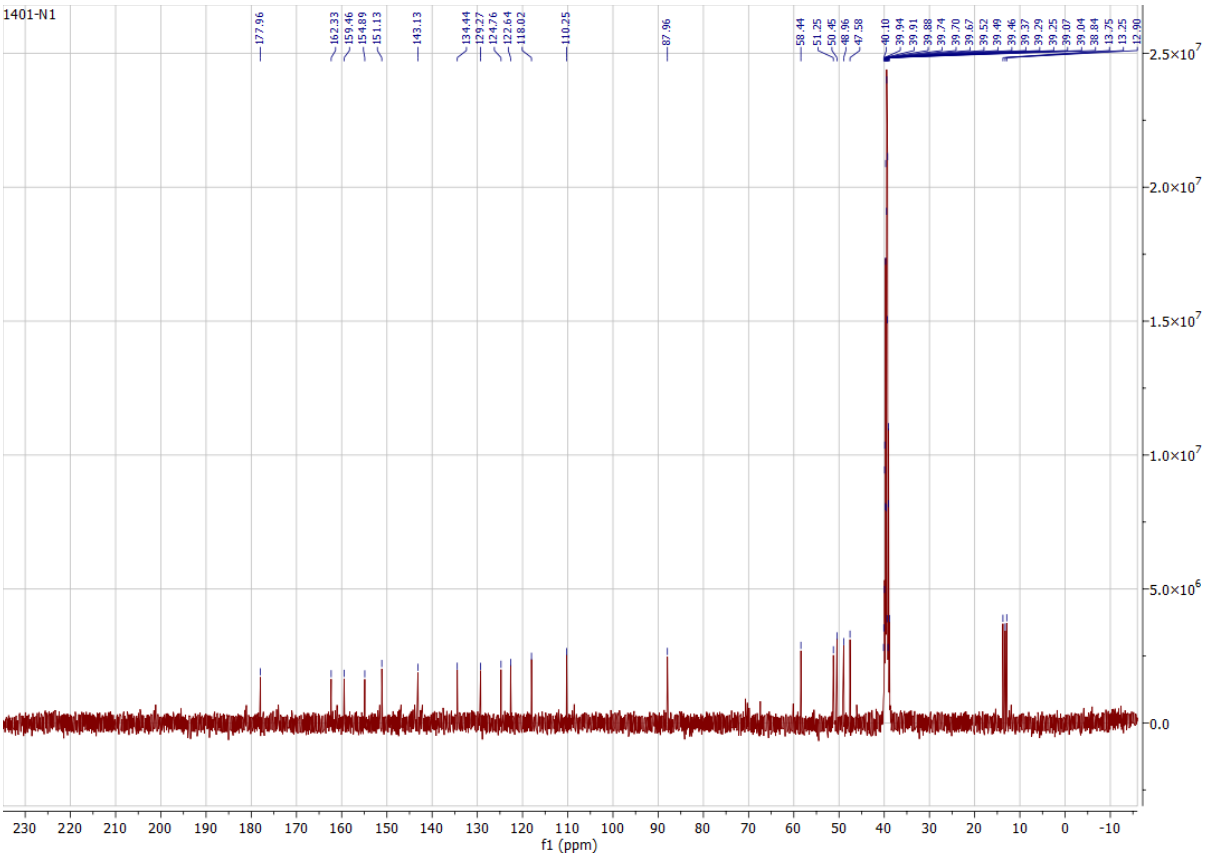

Figure S4. ^{13}C NMR spectrum of 7'-amino-1,1',3'-triethyl-2,2',4'-trioxo-1',2',3',4'-tetrahydrospiro[indoline-3,5'-pyrano[2,3-*d*]pyrimidine]-6'-carbonitrile (**2c**) in DMSO-d6.

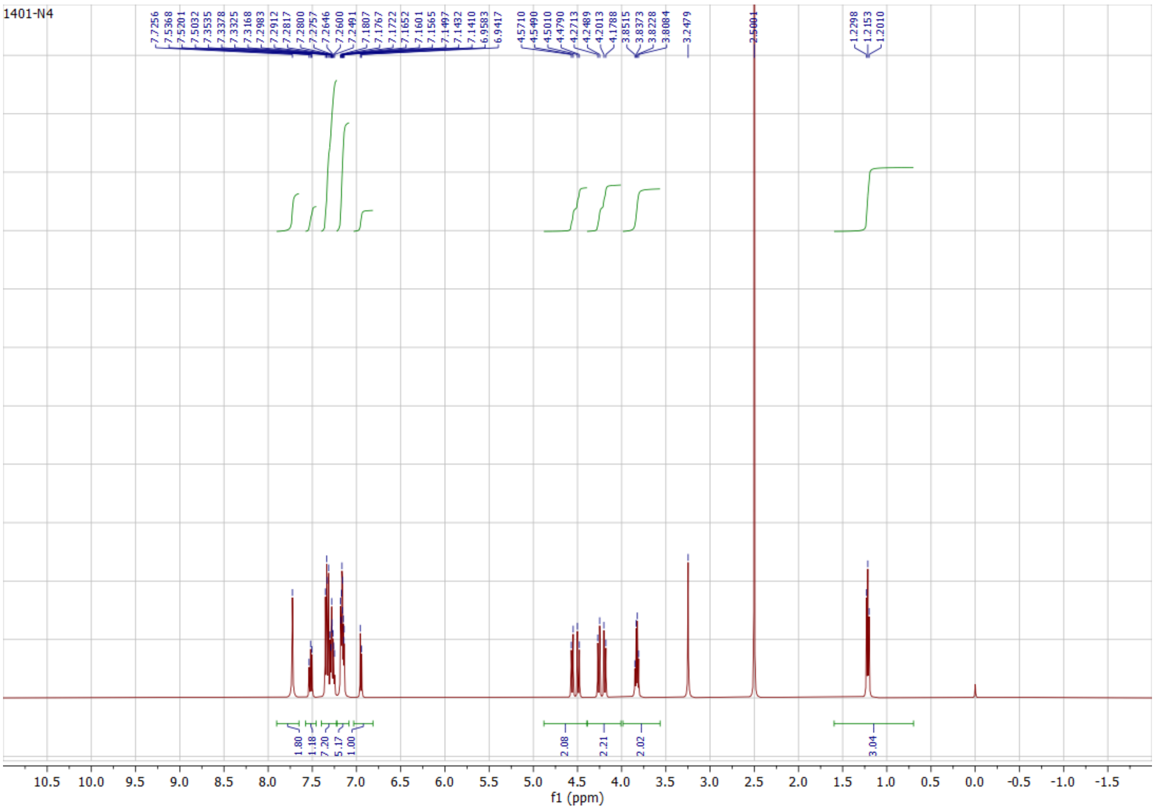

Figure S5. ^{1}H NMR spectrum of 7'-amino-1',3'-dibenzyl-1-ethyl-2,2',4'-trioxo-1',2',3',4'-tetrahydrospiro[indoline-3,5'-pyrano[2,3-*d*]pyrimidine]-6'-carbonitrile (**3c**) in DMSO-d_6.

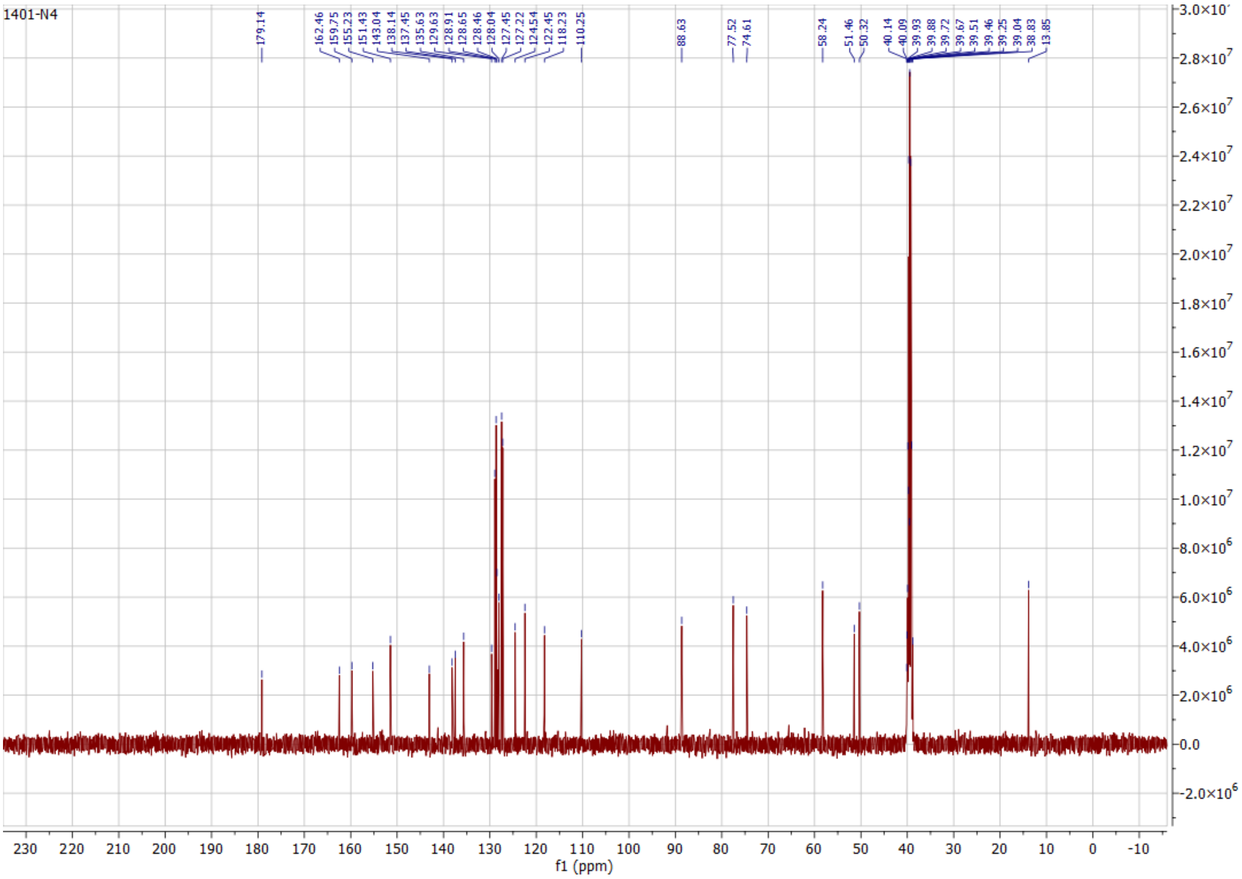

Figure S6. ^{13}C NMR spectrum of 7'-amino-1',3'-dibenzyl-1-ethyl-2,2',4'-trioxo-1',2',3',4'-tetrahydrospiro[indoline-3,5'-pyrano[2,3-d]pyrimidine]-6'-carbonitrile (**3c**) in DMSO-d_6.

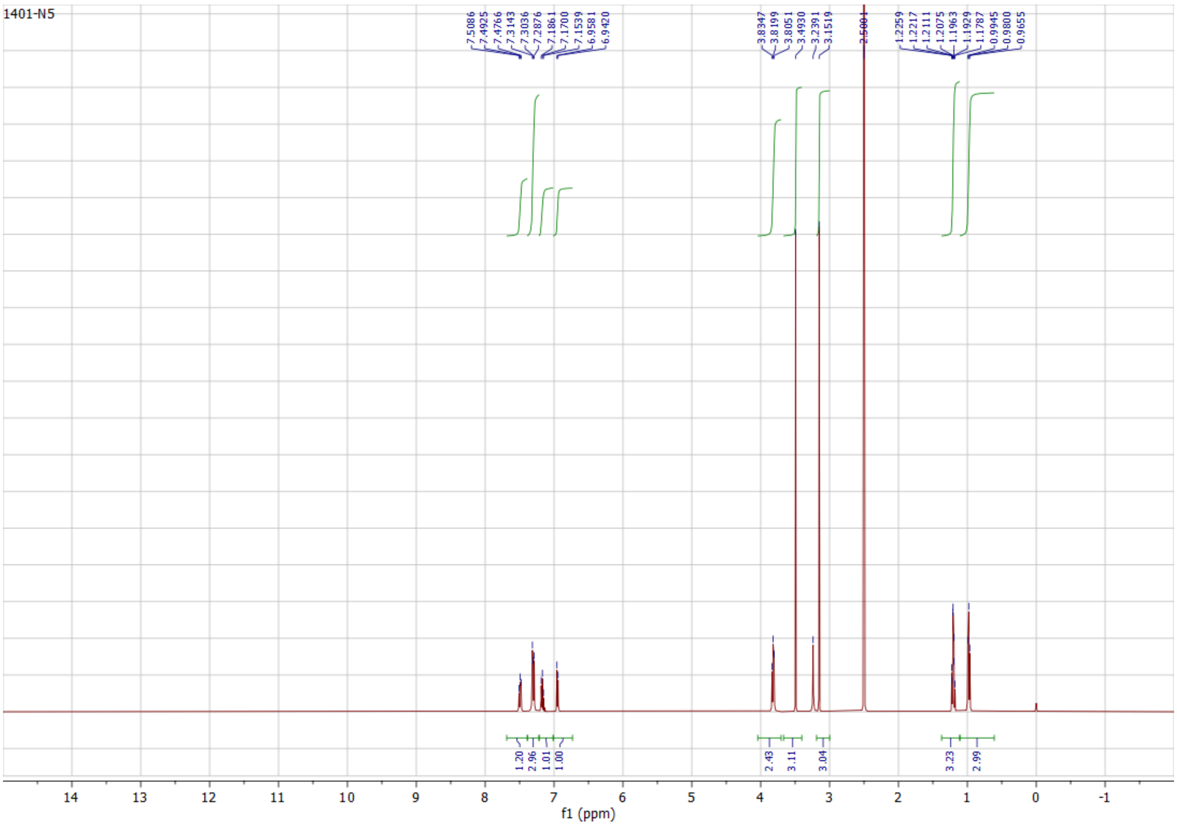

Figure S7. ^{1}H NMR spectrum of 7'-amino-1',3'-dimethyl-2,2',4'-trioxo-1-propyl-1',2',3',4'-tetrahydrospiro[indoline-3,5'-pyrano[2,3-*d*]pyrimidine]-6'-carbonitrile (**4c**) in DMSO-d6.

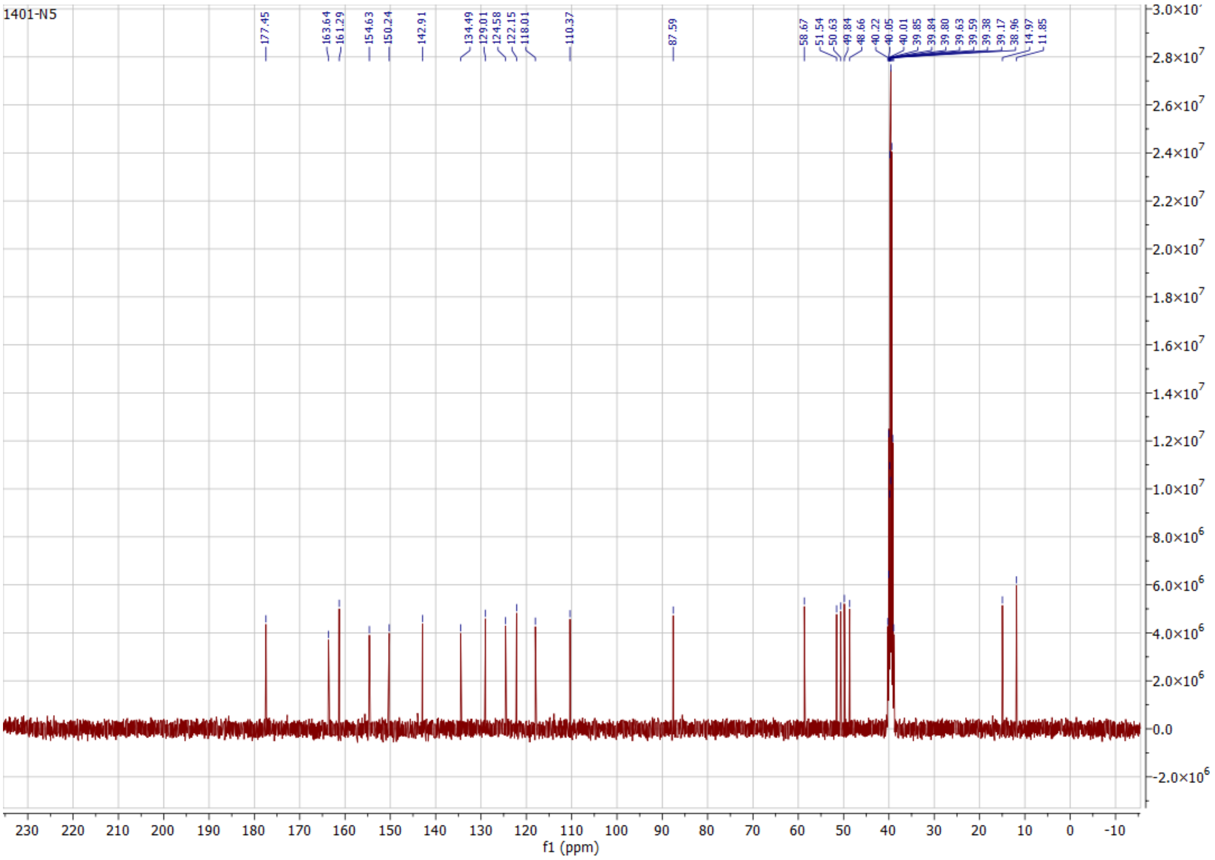

Figure S8. ^{13}C NMR spectrum of 7'-amino-1',3'-dimethyl-2,2',4'-trioxo-1-propyl-1',2',3',4'-tetrahydrospiro[indoline-3,5'-pyrano[2,3-*d*]pyrimidine]-6'-carbonitrile (**4c**) in DMSO-d6.

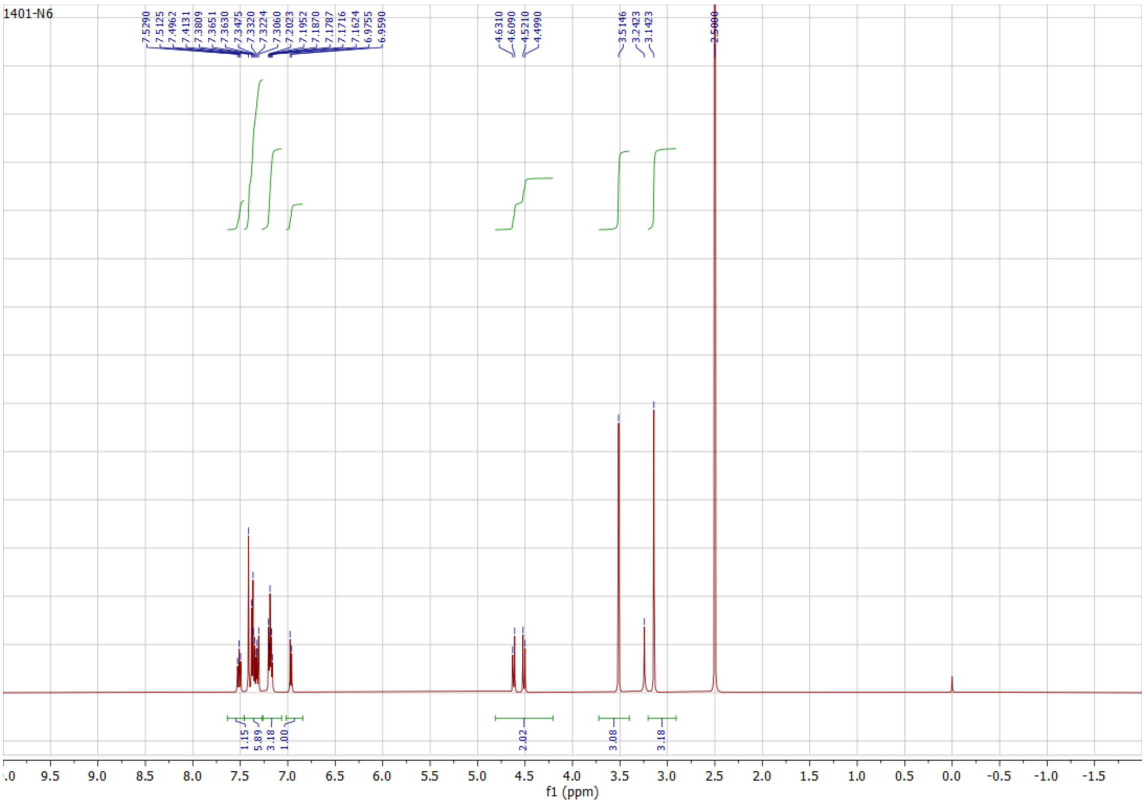

Figure S9. ^{1}H NMR spectrum of 7'-amino-1-benzyl-1',3'-dimethyl-2,2',4'-trioxo-1',2',3',4'-tetrahydrospiro[indoline-3,5'-pyrano[2,3-*d*]pyrimidine]-6'-carbonitrile (**5c**) in DMSO-d6.

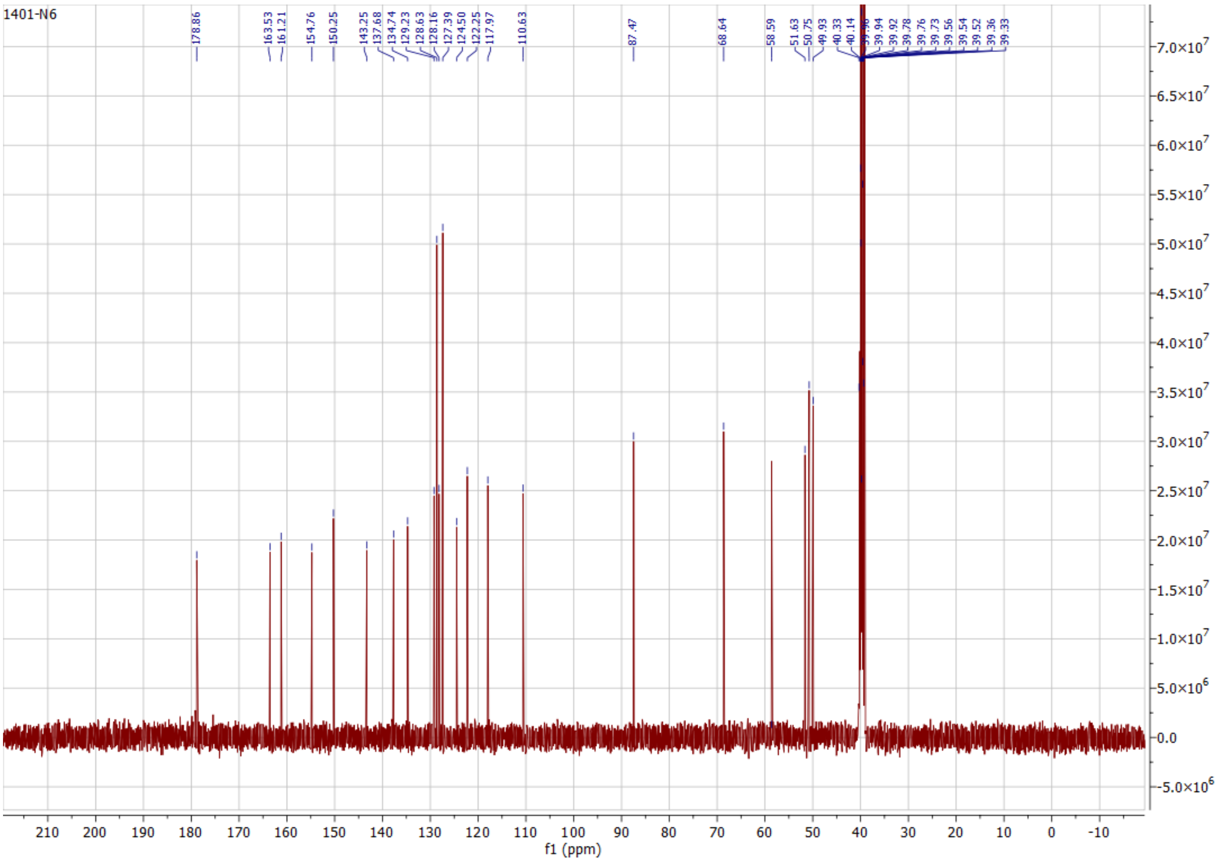

Figure S10. ^{13}C NMR spectrum of 7'-amino-1-benzyl-1',3'-dimethyl-2,2',4'-trioxo-1',2',3',4'-tetrahydrospiro[indoline-3,5'-pyrano[2,3-*d*]pyrimidine]-6'-carbonitrile (**5c**) in DMSO-d6.

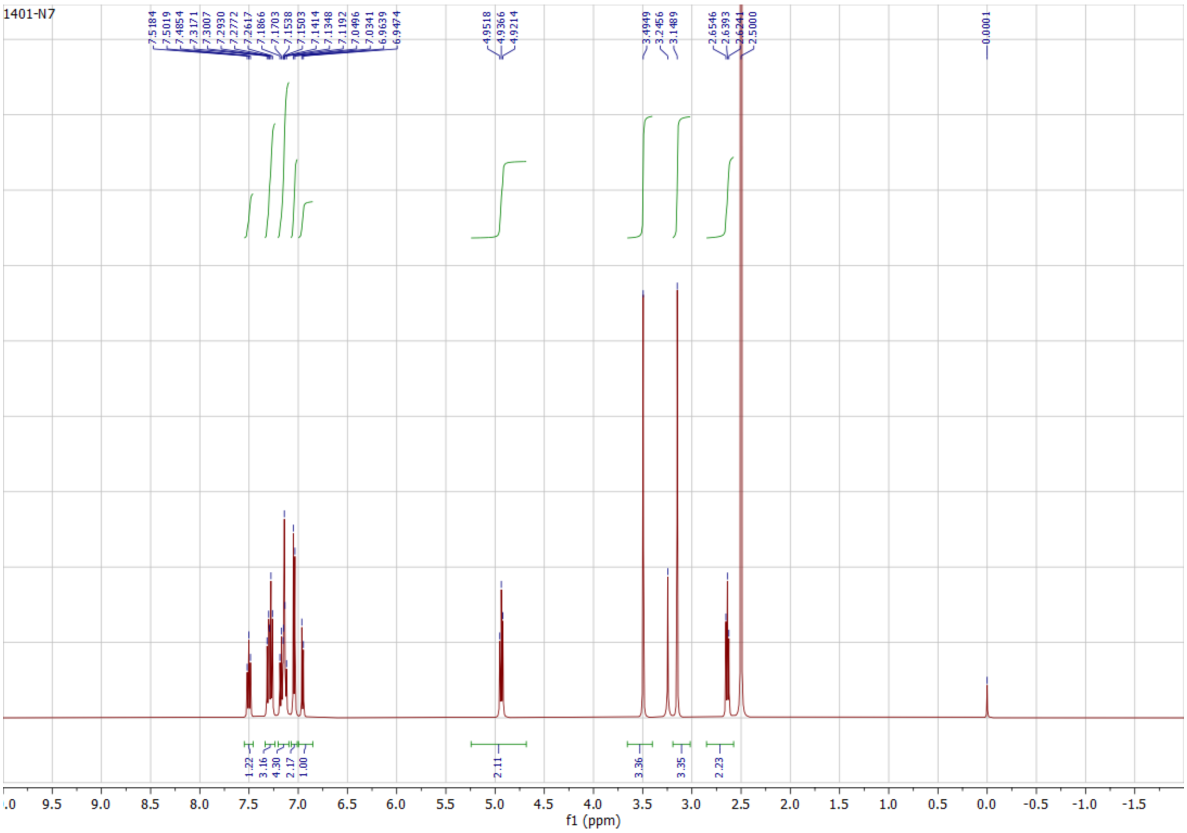

Figure S11. ^{1}H NMR spectrum of 7'-amino-1',3'-dimethyl-2,2',4'-trioxo-1-phenethyl-1',2',3',4'-tetrahydrospiro[indoline-3,5'-pyrano[2,3-*d*]pyrimidine]-6'-carbonitrile (**6c**) in DMSO-d_6.

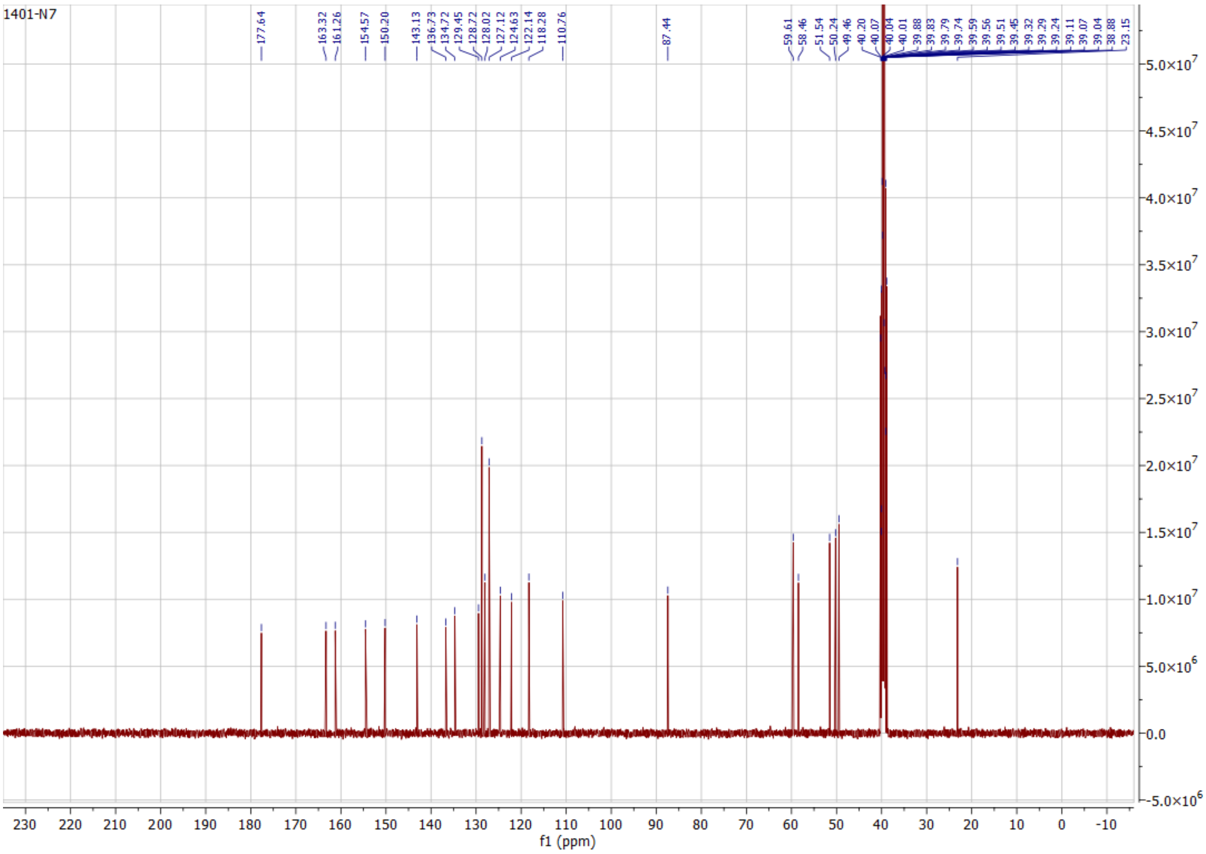

Figure S12. ^{13}C NMR spectrum of 7'-amino-1',3'-dimethyl-2,2',4'-trioxo-1-phenethyl-1',2',3',4'-tetrahydrospiro[indoline-3,5'-pyrano[2,3-*d*]pyrimidine]-6'-carbonitrile (**6c**) in DMSO-d_6.

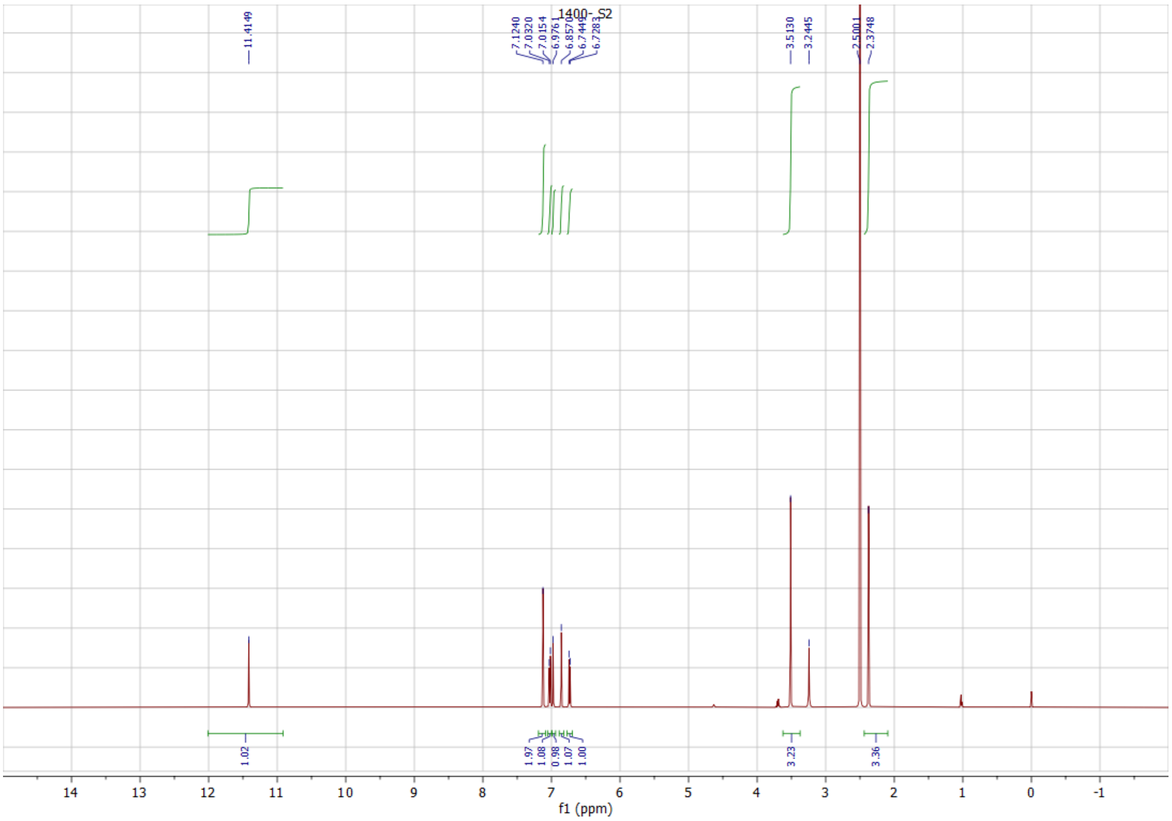

Figure S13. ^{1}H NMR spectrum of methyl 6'-amino-5'-cyano-5-methyl-2-oxo-2'H-spiro[indoline-3,4'-pyrano[2,3-*c*]pyrazole]-3'-carboxylate (**1d**) in DMSO-d6.

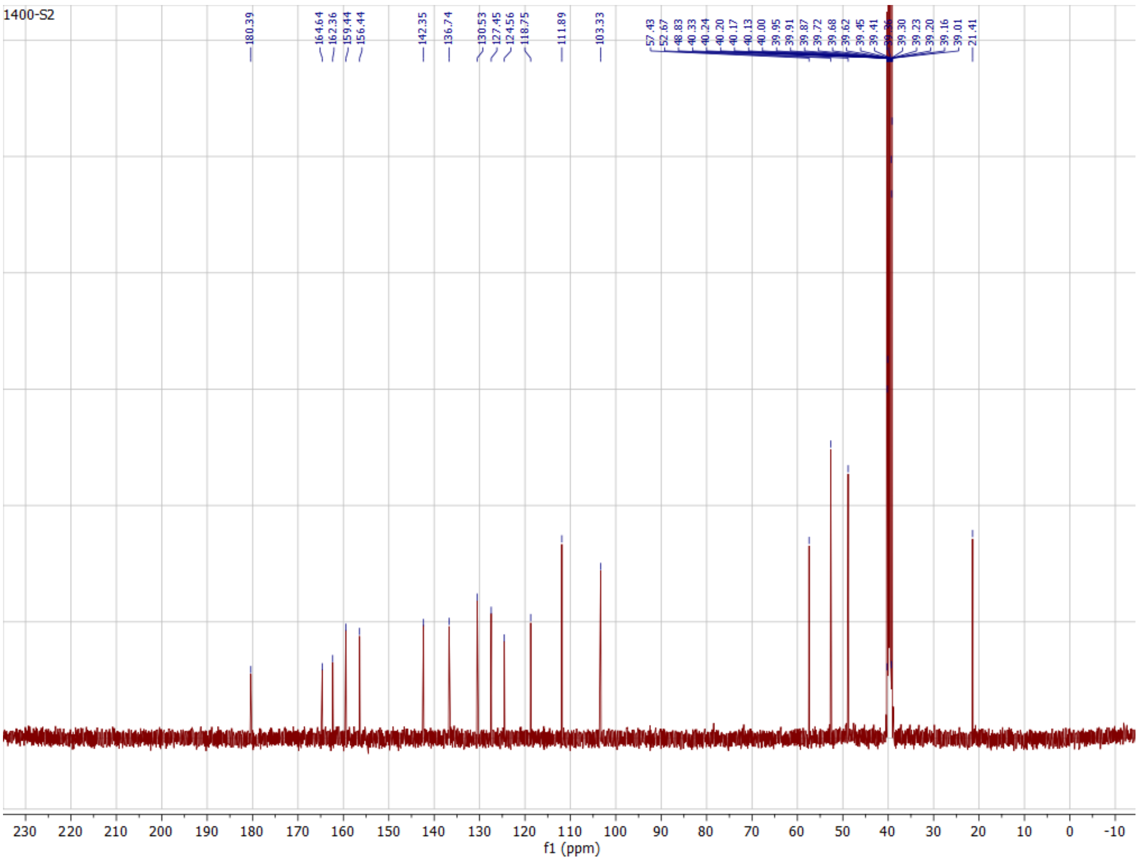

Figure S14. ^{13}C NMR spectrum of methyl 6'-amino-5'-cyano-5-methyl-2-oxo-2'*H*-spiro[indoline-3,4'-pyrano[2,3-*c*]pyrazole]-3'-carboxylate (**1d**) in DMSO-d6.

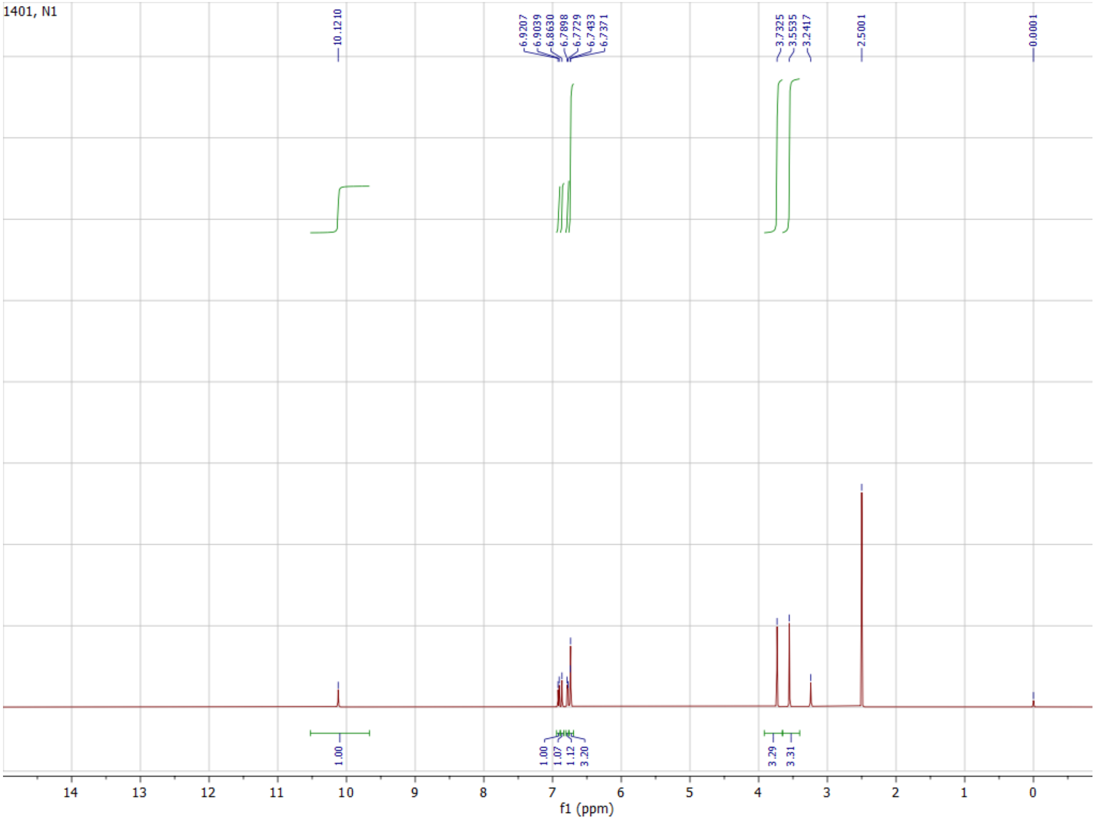

Figure S15. ^{1}H NMR spectrum of methyl 6'-amino-5'-cyano-5-methoxy-2-oxo-2'H-spiro[indoline-3,4'-pyrano[2,3-*c*]pyrazole]-3'-carboxylate (**2d**) in DMSO-d6.

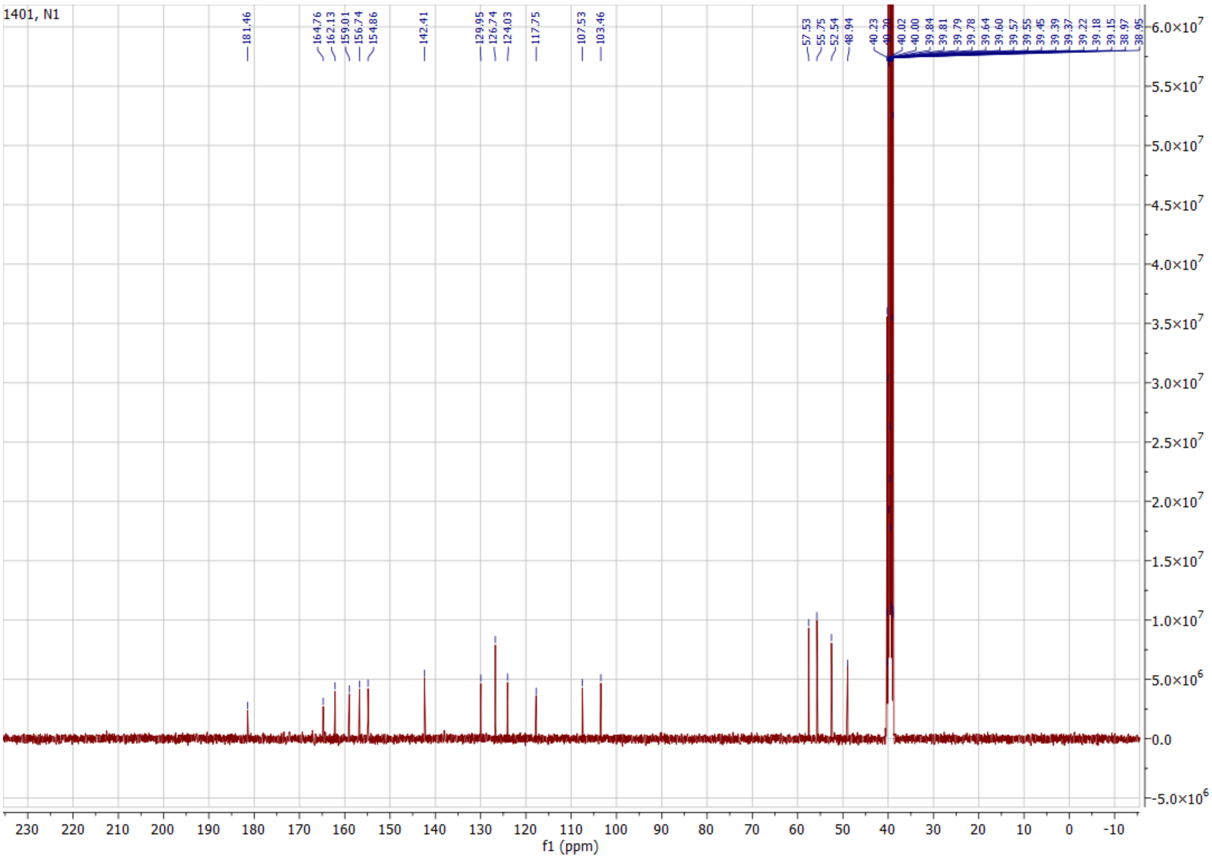

Figure S16. ^{13}C NMR spectrum of methyl 6'-amino-5'-cyano-5-methoxy-2-oxo-2'*H*-spiro[indoline-3,4'-pyrano[2,3-*c*]pyrazole]-3'-carboxylate (**2d**) in DMSO-d6.

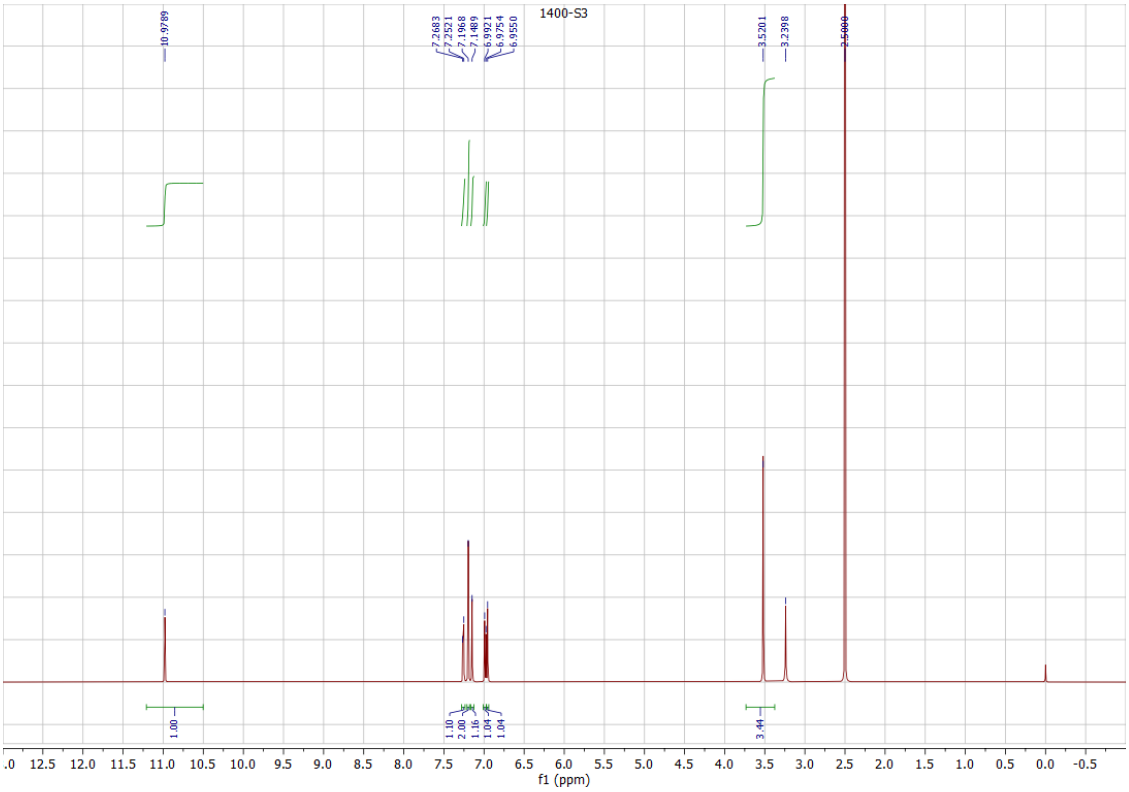

Figure S17. ^{1}H NMR spectrum of methyl 6'-amino-5-chloro-5'-cyano-2-oxo-2'*H*-spiro[indoline-3,4'-pyrano[2,3-*c*]pyrazole]-3'-carboxylate (**3d**) in DMSO-d6.

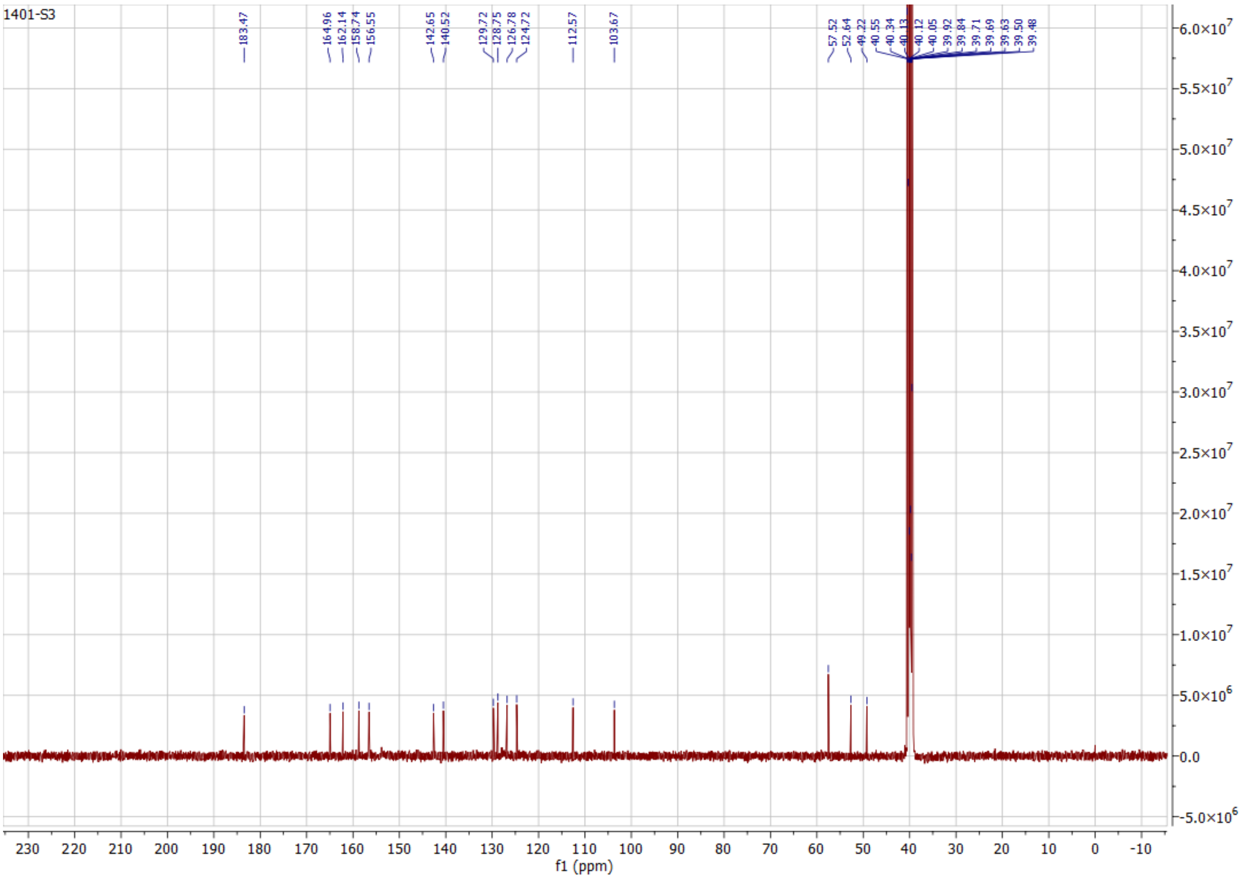

Figure S18. ^{13}C NMR spectrum of methyl 6'-amino-5-chloro-5'-cyano-2-oxo-2'*H*-spiro[indoline-3,4'-pyrano[2,3-*c*]pyrazole]-3'-carboxylate (**3d**) in DMSO-d6.

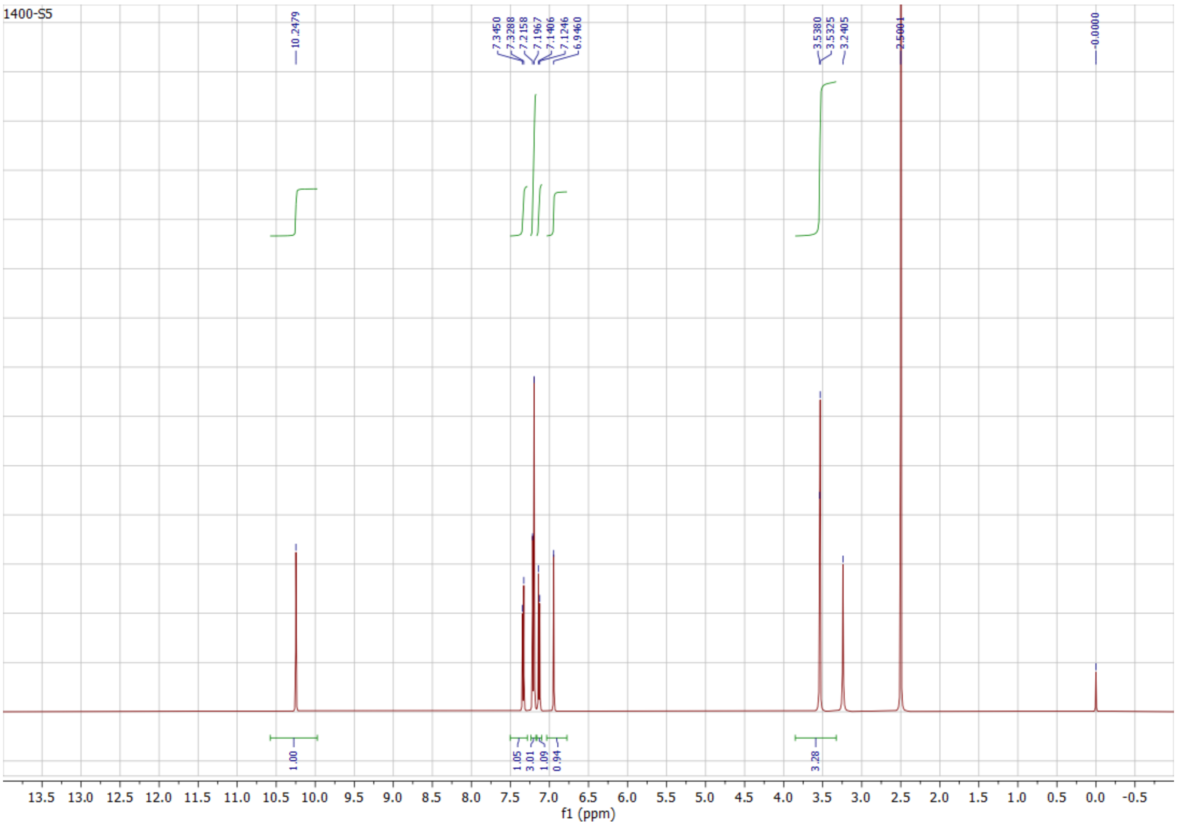

Figure S19. ^{1}H NMR spectrum of methyl 6'-amino-5-bromo-5'-cyano-2-oxo-2'*H*-spiro[indoline-3,4'-pyrano[2,3-*c*]pyrazole]-3'-carboxylate (**4d**) in DMSO-d$_6$.

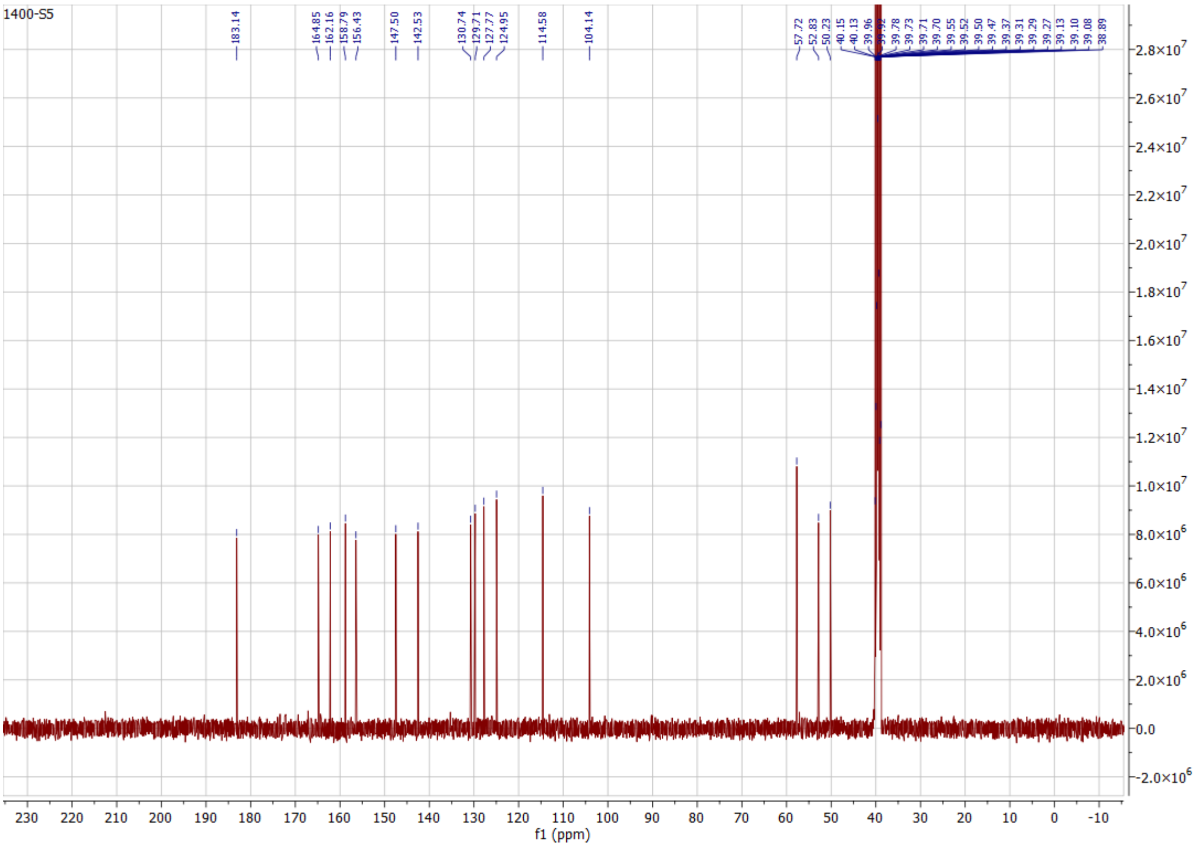

Figure S20. ^{13}C NMR spectrum of methyl 6'-amino-5-bromo-5'-cyano-2-oxo-2'*H*-spiro[indoline-3,4'-pyrano[2,3-*c*]pyrazole]-3'-carboxylate (**4d**) in DMSO-d$_6$.

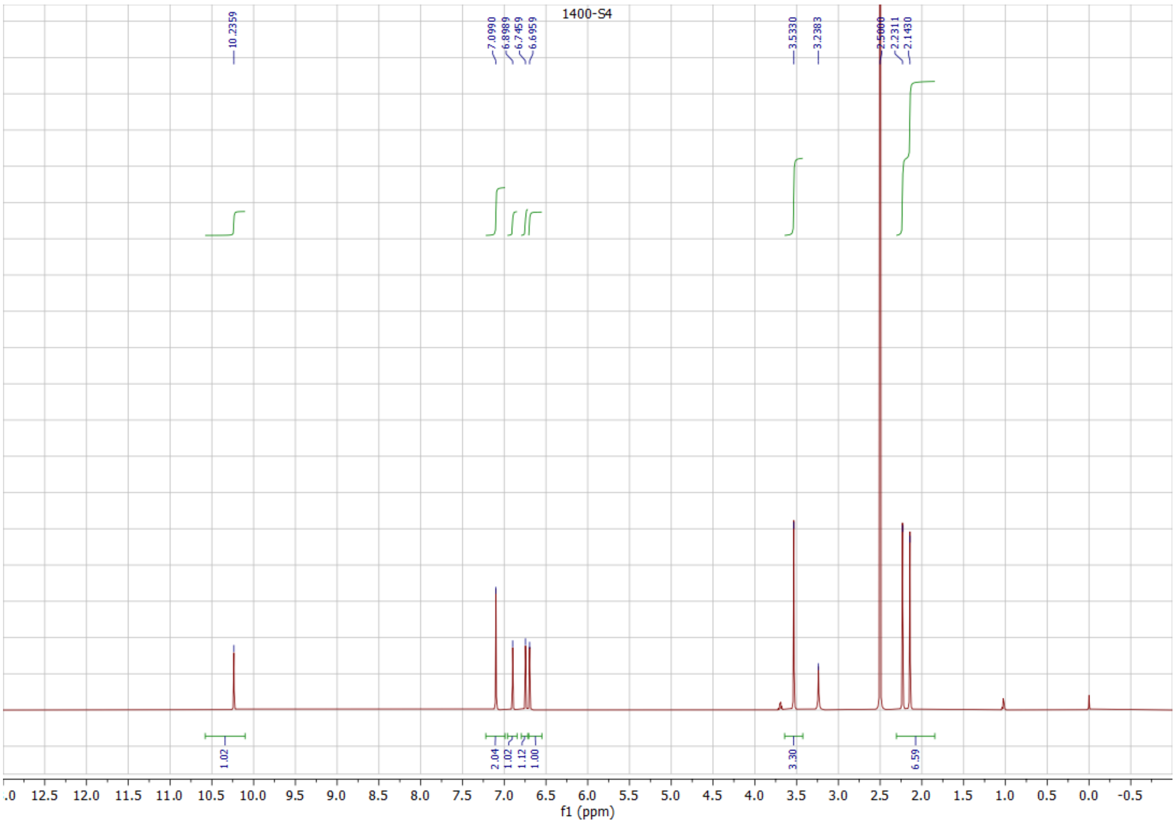

Figure S21. ^{1}H NMR spectrum of methyl 6'-amino-5'-cyano-5,7-dimethyl-2-oxo-2'*H*-spiro[indoline-3,4'-pyrano[2,3-*c*]pyrazole]-3'-carboxylate (**5d**) in DMSO-d6.

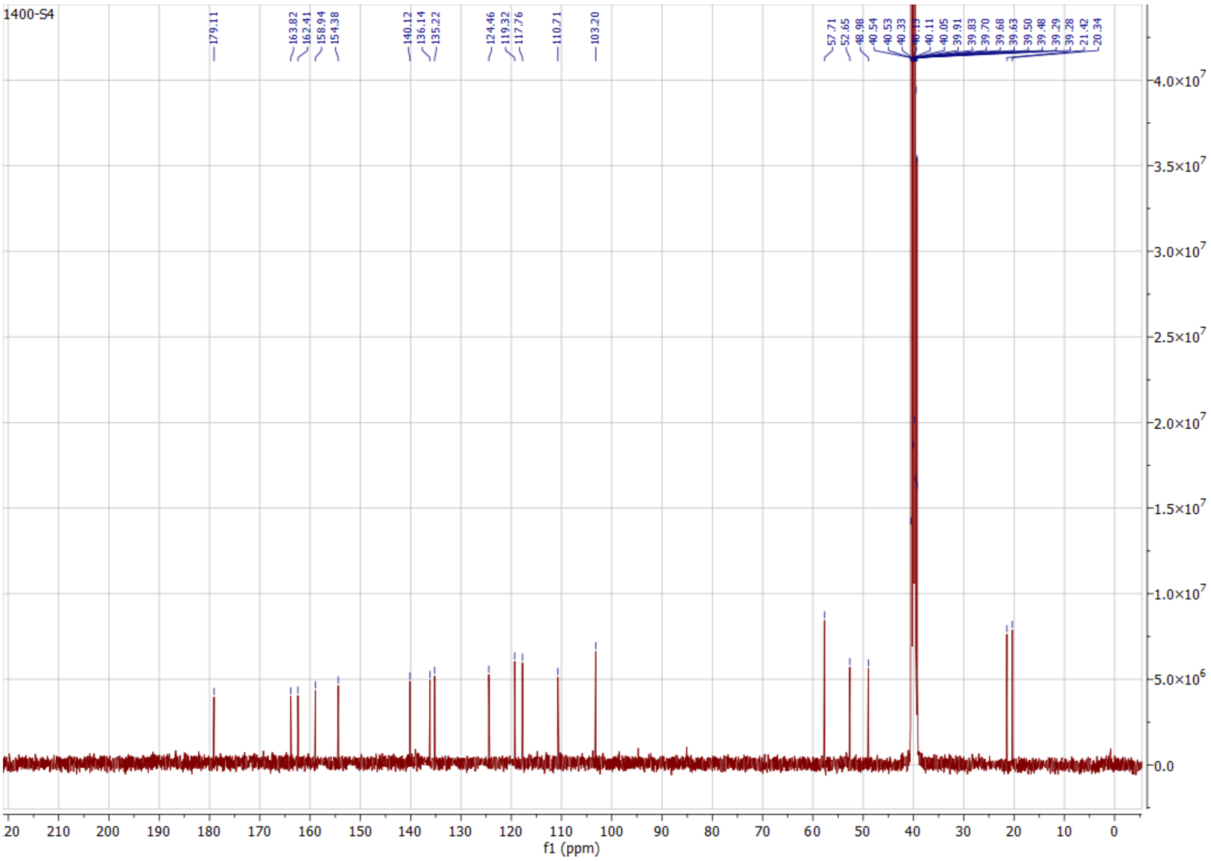

Figure S22. ^{13}C NMR spectrum of methyl 6'-amino-5'-cyano-5,7-dimethyl-2-oxo-2'*H*-spiro[indoline-3,4'-pyrano[2,3-*c*]pyrazole]-3'-carboxylate (**5d**) in DMSO-d6.

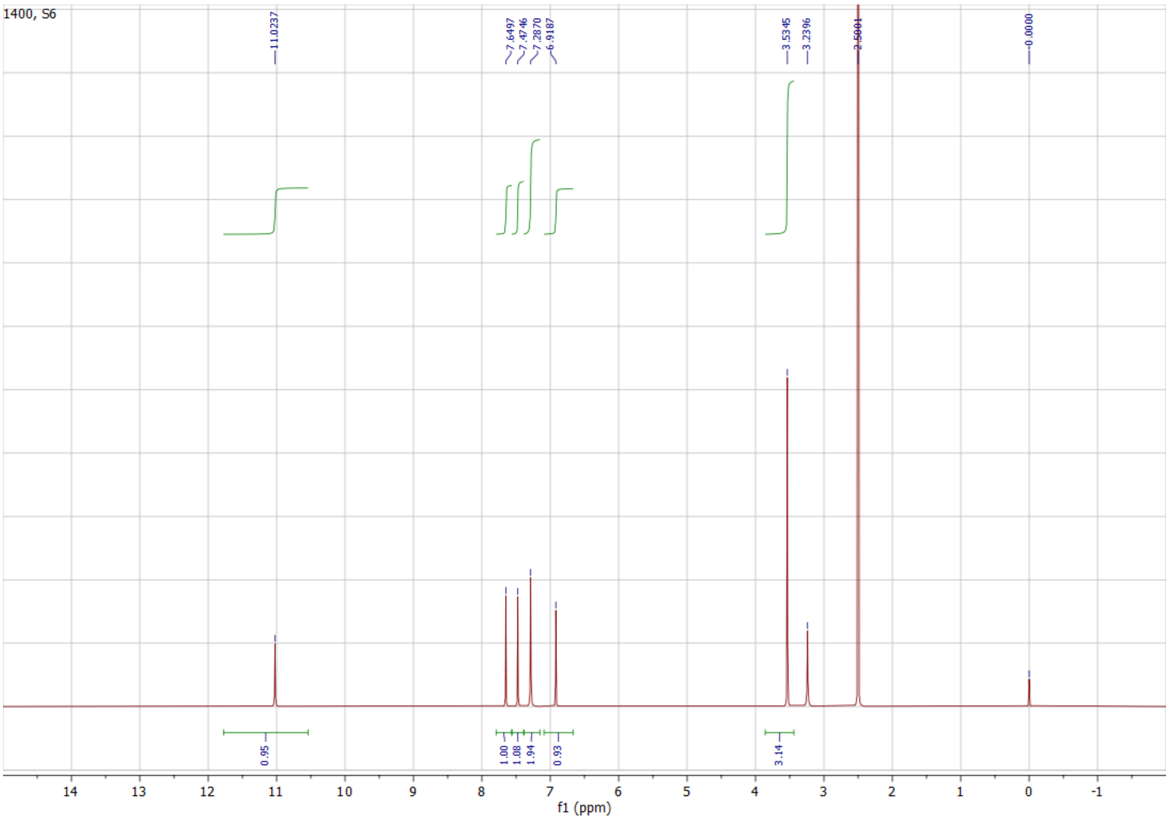

Figure S23. ^{1}H NMR spectrum of methyl 6'-amino-5,7-dibromo-5'-cyano-2-oxo-2'*H*-spiro[indoline-3,4'-pyrano[2,3-*c*]pyrazole]-3'-carboxylate (**6d**) in DMSO-d_6.

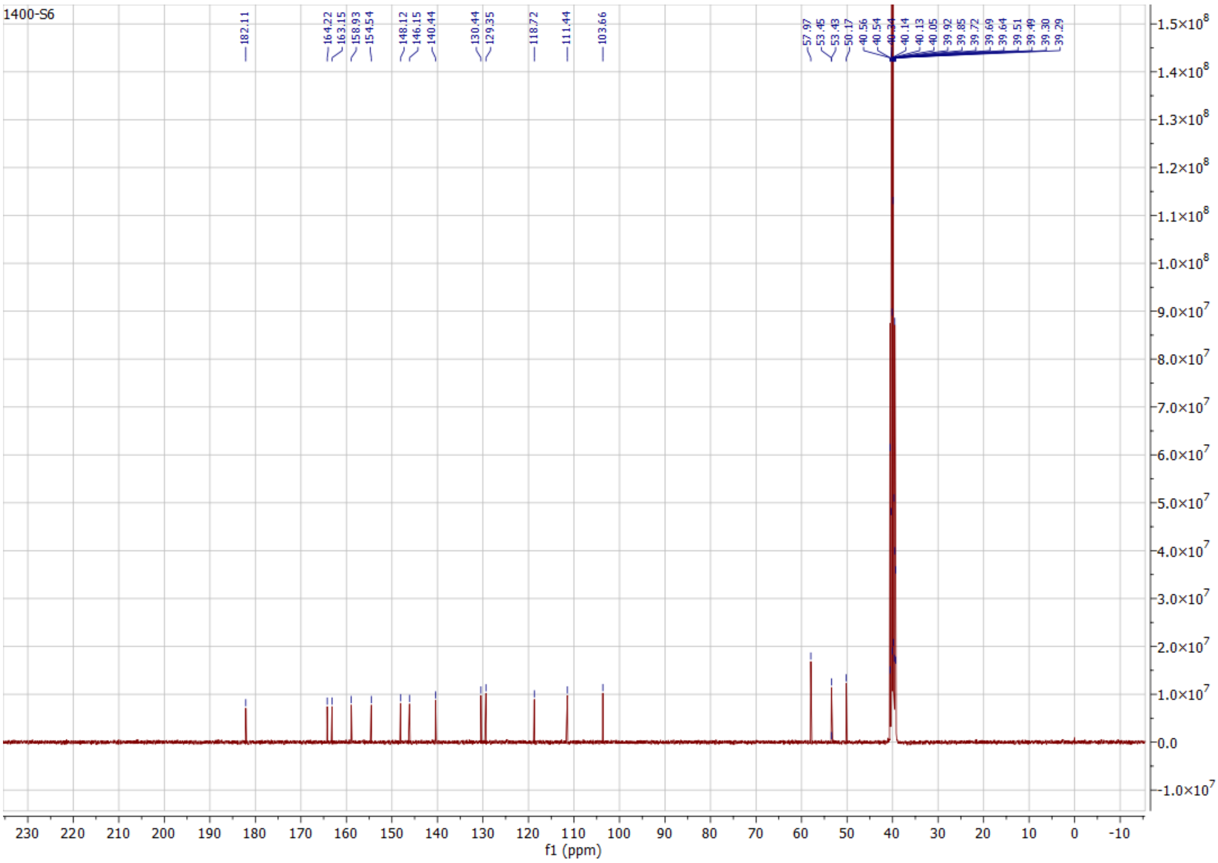

Figure S24. ^{13}C NMR spectrum of methyl 6'-amino-5,7-dibromo-5'-cyano-2-oxo-2'*H*-spiro[indoline-3,4'-pyrano[2,3-*c*]pyrazole]-3'-carboxylate (**6d**) in DMSO-d_6.

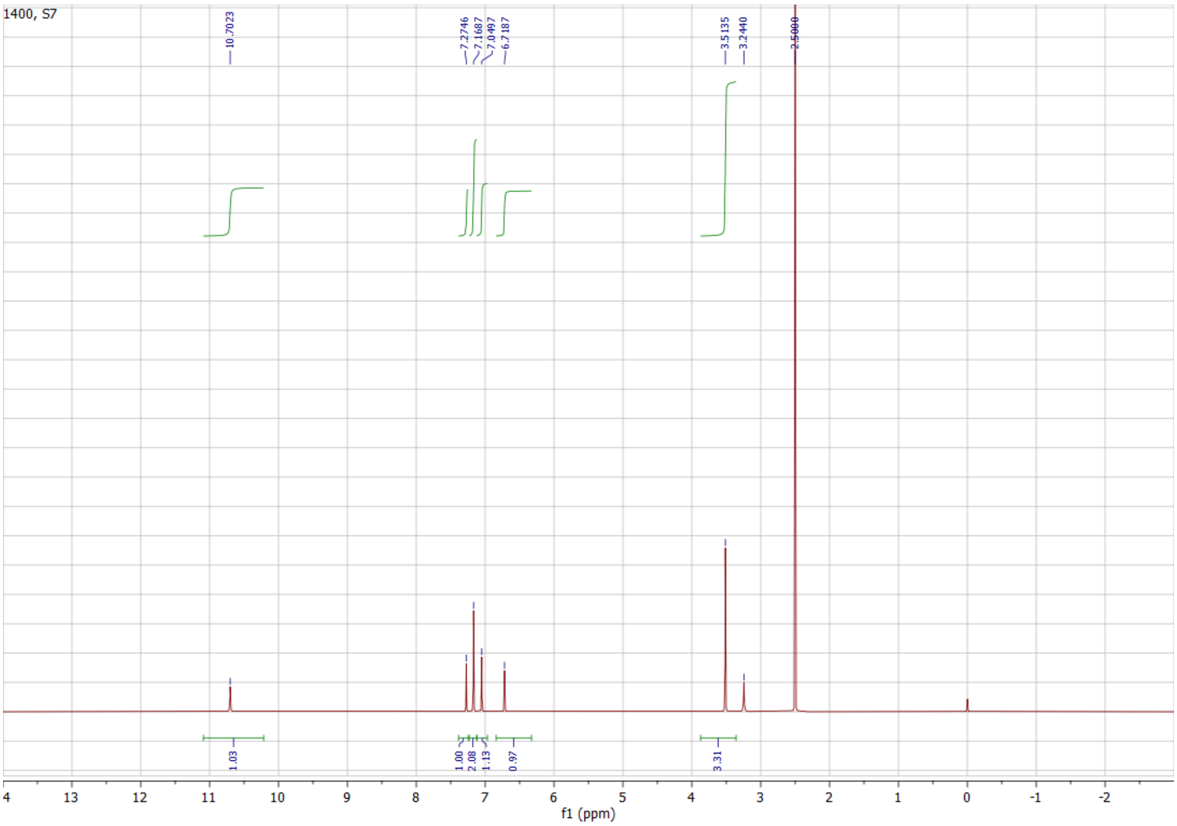

Figure S25. ^{1}H NMR spectrum of methyl 6'-amino-5,7-dichloro-5'-cyano-2-oxo-2'*H*-spiro[indoline-3,4'-pyrano[2,3-*c*]pyrazole]-3'-carboxylate (**7d**) in DMSO-d_6.

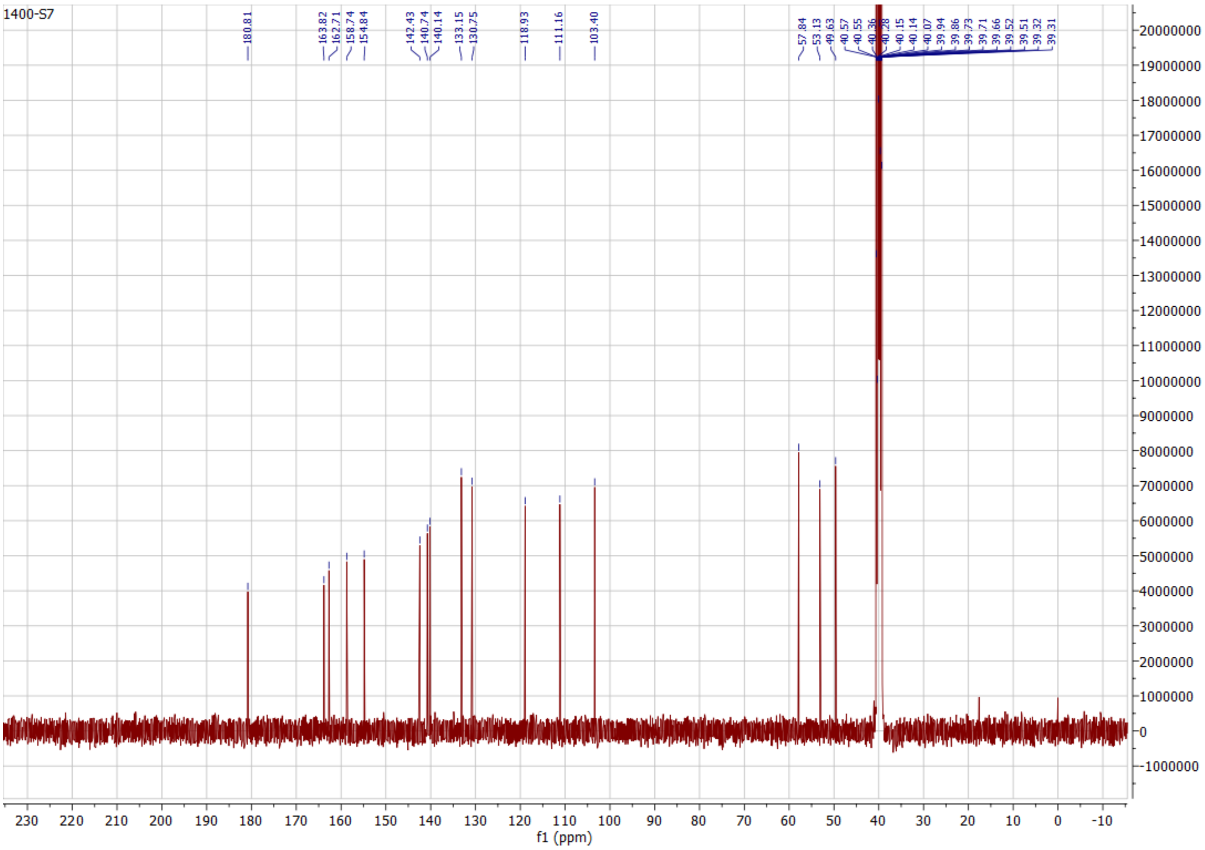

Figure S26. ^{13}C NMR spectrum of methyl 6'-amino-5,7-dichloro-5'-cyano-2-oxo-2'*H*-spiro[indoline-3,4'-pyrano[2,3-*c*]pyrazole]-3'-carboxylate (**7d**) in DMSO-d_6.

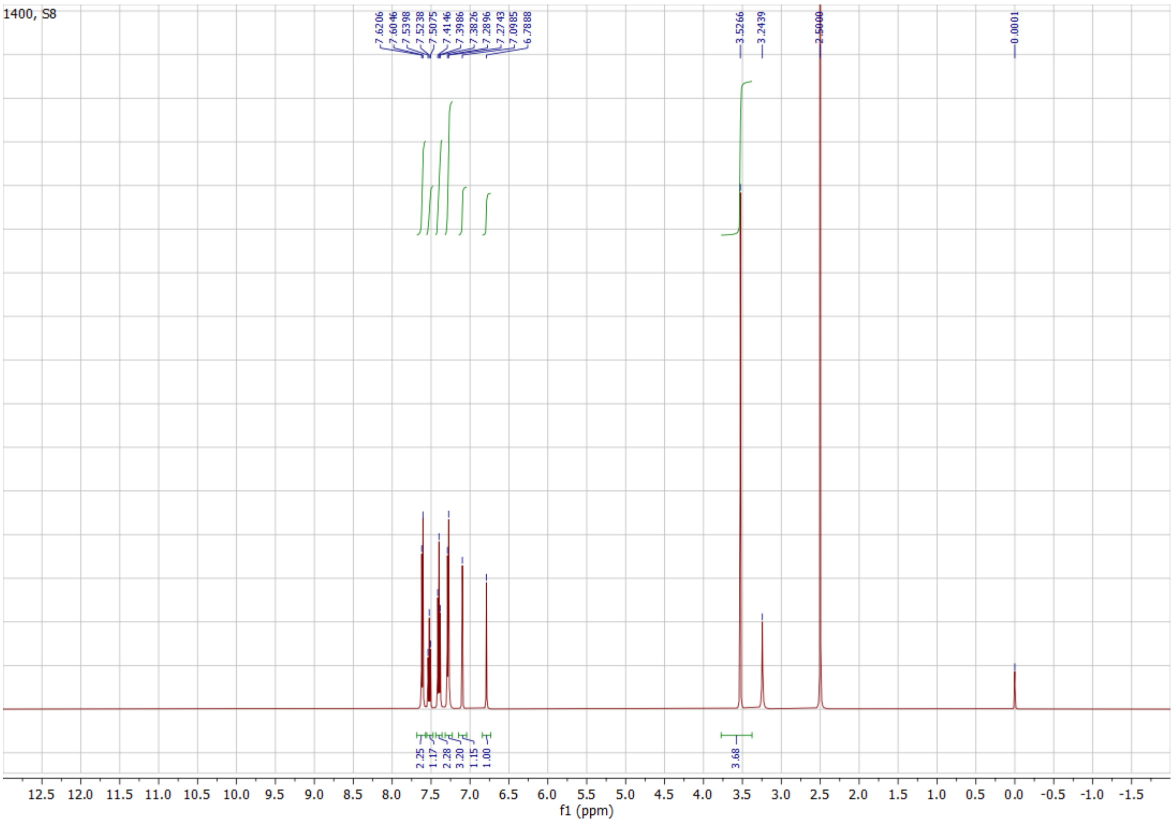

Figure S27. ^{1}H NMR spectrum of methyl 6'-amino-5,7-dichloro-5'-cyano-2-oxo-1-phenyl-2'*H*-spiro[indoline-3,4'-pyrano[2,3-*c*]pyrazole]-3'-carboxylate (**8d**) in DMSO-d_6.

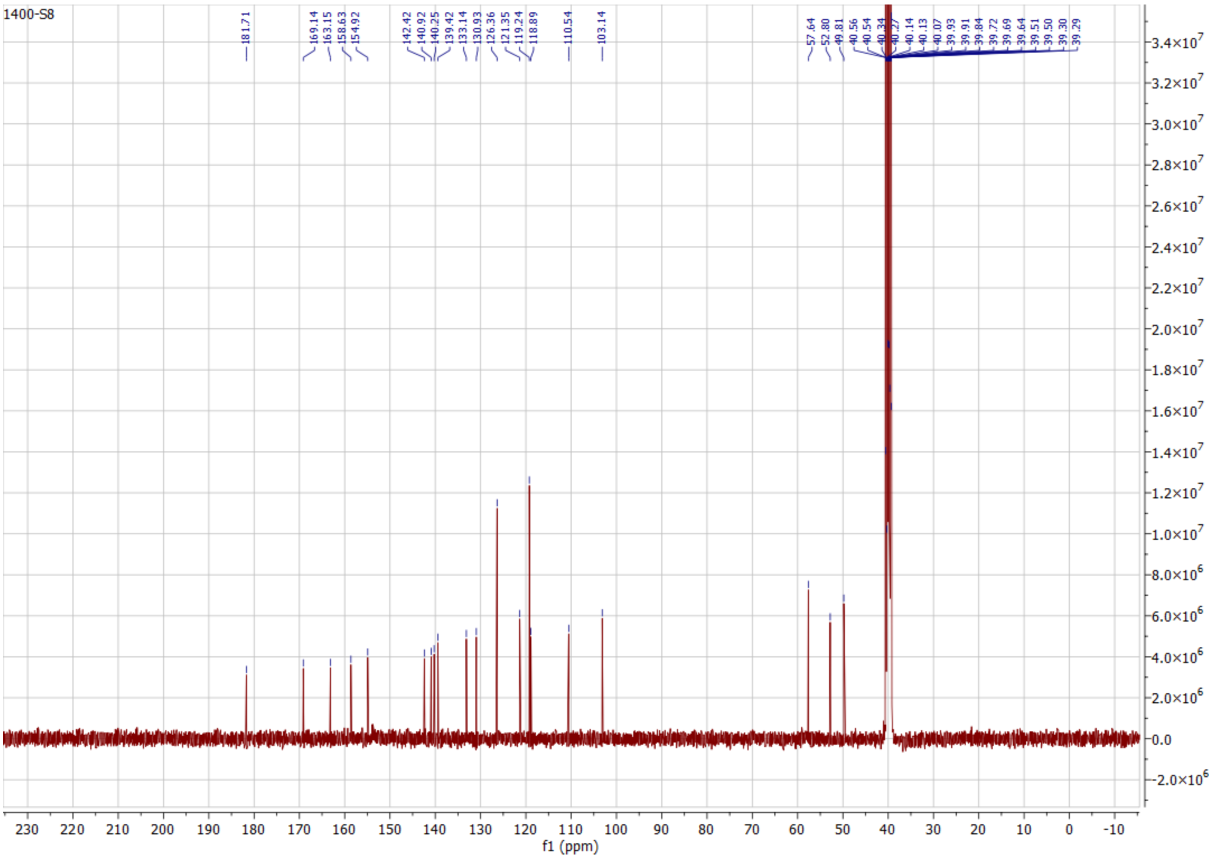

Figure S28. ^{13}C NMR spectrum of methyl 6'-amino-5,7-dichloro-5'-cyano-2-oxo-1-phenyl-2'*H*-spiro[indoline-3,4'-pyrano[2,3-*c*]pyrazole]-3'-carboxylate (**8d**) in DMSO-d_6.
